# Impact of propionic acid-rich diets on microbial composition of the murine gut microbiome

**DOI:** 10.3389/frmbi.2024.1451735

**Published:** 2024-10-22

**Authors:** Noah Greenman, Latifa S. Abdelli, Sayf Al-Deen Hassouneh, Sobur Ali, Catherine Johnston, Saleh A. Naser, Taj Azarian

**Affiliations:** ^1^ Burnett School of Biomedical Sciences, College of Medicine, University of Central Florida, Orlando, FL, United States; ^2^ Department of Health Sciences, College of Health Professions and Sciences, University of Central Florida, Orlando, FL, United States

**Keywords:** third-generation sequencing, nanopore sequencing, gut microbiome, propionic acid, metagenomics, dysbiosis

## Abstract

Propionic acid (PPA), an anti-fungal agent and common food additive, has been shown to induce atypical neurodevelopment in mice, accompanied by gastrointestinal dysfunction potentially resulting from gut dysbiosis. A putative association between dietary PPA exposure and gut dysbiosis is suggested but has not been explored directly. Here, we investigated PPA-associated alteration in gut microbial composition that may result in dysbiosis. Using long-read metagenomic sequencing, gut microbiomes of mice fed an untreated (n=9) or PPA-rich (n=13) diet were sequenced to assess differences in microbial composition and bacterial metabolic pathways. Dietary PPA was associated with an increased abundance of notable taxa, including several species of *Bacteroides, Prevotella*, and *Ruminococcus*, whose member species have previously been associated with PPA production. Microbiomes of PPA exposed mice also possessed a greater abundance of pathways related to lipid metabolism and steroid hormone biosynthesis. Our findings demonstrate PPA’s effect in altering the gut microbiota and associated metabolic pathways. These observed changes highlight how preservatives listed as safe for consumption may affect gut microbiome composition with implications for one’s health.

## 1 Introduction

Often referred to as the “last human organ”, the human microbiome plays an integral role in one’s health (Baquero and Nombela, 2012). In particular, the gut microbiome is well recognized for its system-wide influence and role in several key functions. Commensal organisms are prolific in the gut, occupying multiple niches, utilizing nutrients, and outcompeting potential pathogens (Jandhyala et al., 2015). Several bacterial constituents of the gut microbiome produce essential nutrients such as vitamins and assist with digestion (Rowland et al., 2018). Bacterially-produced metabolites have also been shown to influence the development of tissues as well as augment metabolic and immune-related pathways (Heijtz et al., 2011; Yu et al., 2022). The composition of the human gut microbiome is extremely diverse, shaped by both genetic and environmental factors such as diet, sex, medication, and health conditions (Kumbhare et al., 2019).

Maternal diet is a key component of prenatal and neonatal development and a putative source of exposure to compounds that can impact development (Bazer et al., 2004; Innis, 2014). Propionic acid (PPA), a short-chain fatty acid byproduct of bacterial fermentation and food additive, is one such compound of interest (den Besten et al., 2013). PPA exhibits antimicrobial and antifungal properties, for which it is used as a food preservative and to inhibit mold and bacterial growth in industrial applications (Wemmenhove et al., 2016). PPA exhibits variable effects depending on the tissue. In the liver, PPA exerts an anti-inflammatory effect by targeting expression of cytokines from macrophages (Kawasoe et al., 2022). This modulatory effect has also been observed on other immune cells resulting in down-regulation of inflammation (Haase et al., 2021). However, in the brain, a contrasting effect is observed. Previous work has shown that PPA exposure induced ASD-like behaviors in mice (El-Ansary et al., 2012). Other research suggests PPA may induce neural gliosis and upregulate pro-inflammatory pathways in the brain (Abdelli et al., 2019). Because PPA is a weak acid, it can diffuse across the intestinal epithelium into circulation, allowing it to cross restrictive barriers, including the blood brain barrier, as well as the placenta (Stinson et al., 2019), highlighting PPA’s importance as a bacterially-produced regulatory metabolite. While PPA’s potential role as an environmental risk factor for ASD is currently under investigation, its effects in those with ASD may go beyond induction of neurodivergence.

Gastrointestinal symptoms including diarrhea and constipation are common commodities among individuals with neurodevelopmental disorders (Cao et al., 2021). Previous work has shown that individuals with ASD exhibit different microbiomes from healthy counterparts, suggesting gut dysbiosis (Finegold et al., 2010). Similarly, individuals with conditions such as inflammatory bowel diseases, obesity, Alzheimer’s disease, among others, were shown to have distinct microbiome profiles compared to healthy individuals (Turnbaugh et al., 2009; Vogt et al., 2017; Henke et al., 2019). However, to date, no causal association between the gut microbiome and neurological conditions or symptoms has been established (Yap et al., 2021), though several species are implicated in playing a role in several of these disease states. For example, genera such as *Akkermansia, Bacteroides, Clostridium, Lactobacillus, Desulfovibrio*, and others have been found in greater abundance in microbiomes of those with ASD (Tomova et al., 2015; Golubeva et al., 2017; Cristiano et al., 2018; Zurita et al., 2020). Notably, several of these genera contain member species known to possess genes associated with production of PPA (Reichardt et al., 2014; Yun and Lee, 2016; Zhang et al., 2019; Baur and Dürre, 2023). Given its antimicrobial nature, increased levels of PPA may play a role in favoring PPA producers (Jacobson et al., 2018). Consequently, PPA rich environment may be responsible for altering the gut microbiome, including gastrointestinal pathogens, which may serve as potential contributors to gastrointestinal symptoms.

A central question in microbiome research is whether differences in composition contribute to, or are a symptom of, the disease state in question. A first step in elucidating the complex relationship between diet, the gut microbiome, and neurological conditions is to assess the directional impact of diet on microbial composition. Toward this end, we used long-read metagenomic sequencing to compare the gut microbiomes of progeny mice from mothers provided food replete or absent PPA. Progeny mice were maintained on the same diet as their mothers. We hypothesized that PPA-rich diets would result in changes in gut microbial composition and microbial functional pathways, particularly those associated with PPA metabolism and/or PPA production.

## 2 Materials and methods

### 2.1 Animal conditions and fecal extraction

FVB/N-Tg(GFAP-GFP)14Mes/J transgenic mice overexpressing green fluorescent protein (GFP) under the control of the glia-specific GFAP promoter (Jackson Laboratories) were used in this study following the University of Central Florida Institutional Animal Care and Use Committee (UCF-IACUC) guidelines (Animal Use Approval #: PROTO202000002). After weaning, mice were separated into cages with each cage containing 1-5 mice of the same sex. Mice were given *ad libitum* either a purified control diet (Modified Open Standard Diet with 16 kcal% fat) or a sodium propionate-rich diet (Modified Open Standard Diet with 16 kcal% fat with 5000 ppm of sodium propionate). The quantity of sodium propionate given corresponds to 5000 mg PPA per kilogram of total food. This is the maximum PPA concentration allowable for use in the food industry as a preservative. In preparation for this study, parent mice were exposed to either diet 4 weeks prior to mating and continued throughout mothers ‘pregnancy. Progeny mice [Twenty-two mice, 9 control (6 male, 3 female) and 13 PPA (4 male, 9 female)] were weaned off their mothers and then continued the same diet provided to their respective mothers for an additional 5 months. At 5 months of age, progeny mice were sacrificed, at which point fecal contents were collected from the intestines and initially stored at -20°C in 1.5 mL microcentrifuge tubes, then transferred to a -80°C freezer until host DNA depletion and microbial nucleic acid extraction.

### 2.2 Host DNA depletion and bacterial DNA extraction

Host DNA was depleted according to a modified protocol from (Charalampous et al., 2019). Briefly, fecal contents were transferred to 500 µL of InhibitEX (Qiagen, Cat No./ID: 19593) while still frozen. At most, 1-2 pellets of stool were processed for each extraction. Fecal contents were then homogenized mechanically using a plastic pestle within the tube until a slurry formed. Samples were spun at 10,000 RCF for 5 minutes or until the sample had pelleted, after which the supernatant was aspirated, and the pellets were resuspended in 250µL of 1X PBS. Two-hundred and fifty microliters of a solution of 4.4% saponin (TCI, Product No.: S0019) was added to the samples as a detergent to weaken eukaryotic cell membranes. Samples were mixed by gentle agitation and incubated at room temperature for 10 minutes. Next, to break eukaryotic cells, 350µL of nuclease-free water was added to the samples which incubated for 30 seconds, and then 12 µL of 5M NaCl was added. Samples were then spun at 6000 RCF for 5 minutes. The supernatant was aspirated off, and the pellets were resuspended in 100 µL 1X PBS. To remove host DNA, 100 µL of HL-SAN buffer (12.8568 g NaCl, 4 mL 1M MgCl_2_, 36 mL nuclease-free H_2_O) and 10 µL of HL-SAN enzyme (ArticZymes P/N 70910-202) were added. Samples were mixed by pipetting and incubated at 37°C, 800 RPM on an Eppendorf™ ThermoMixer C for 30 minutes. After incubation, samples were spun down at 6000 RCF for 3 minutes, then two washes using first 800 µL, then 1000 µL of 1X PBS were performed. The pellets were then resuspended in 100 µL of 1X PBS.

Total bacterial DNA extraction was performed using the New England Biolabs Monarch Genomic DNA Purification Kit (New England Biolabs, Ipswich, MA, Catalog No. T3010L). The standard protocol provided for the kit was used with some modifications. Before beginning, nuclease-free water was incubated and kept at 60°C for the final elution. Ten microliters of Proteinase K and 3 µL of RNase A were added to each sample. Next, 100 µL of cell lysis buffer was added and mixed by gentle agitation. Samples were then incubated at 56°C, 1400 RPM for at least 1 hour and up to 3 hours on an Eppendorf™ ThermoMixer C. Incubated samples were spun at 12,000 RCF for 3 minutes, and then the supernatant for each sample was transferred to a separate 1.5 mL microcentrifuge tube that contained 400 µL of binding solution. Tubes were then pulse-vortexed for 5-10 seconds at 1 second intervals. All liquid contents (approximately 600 µL-700 µL) for each sample were transferred to filter columns placed in a flowthrough collection tube. Initial binding of DNA was achieved by spinning the tubes at 1000 RCF for 3 minutes before pulling all remaining liquid down by spinning at 12,000 RCF for 1 minute. Sample columns were transferred to new collection tubes, after which two washes were performed. For the first wash, 500 µL of wash buffer were added to each tube. Tubes were inverted 3-5 times prior to being spun at 12,000 RCF for 1 minute. The liquid was removed from the collection tube and then the column was returned to the same collection tube. For wash 2, 500 µL of wash buffer were added to the filter, however no inversions were performed. Samples were spun at 12,000 RCF for 1 minute. Filters were transferred to 1.5 mL LoBind^®^ tubes and 100 µL of the pre-heated nuclease free water was added. Filters were incubated for 1 minute at RT before being spun at 12,000 RCF for 1 minute. Eluted DNA was stored at -80°C.

### 2.3 Library preparation and sequencing

Quantification of DNA concentration was measured using a Qubit™ 4.0 Fluorometer. DNA was prepared using the Qubit™ 1X dsDNA High Sensitivity kit according to the manufacturer’s instructions (Cat. No.: Q33231). DNA fragment length distributions were measured using either the Aglient™ 4150 or 4200 TapeStation. DNA was prepared using the Agilent™ Genomic DNA Reagents (Ref. No.: 5067-5366) and a Genomic DNA ScreenTape (Ref. No.: 5067-5365). Library preparation was performed using the Oxford Nanopore Technologies™ (ONT) Rapid PCR Barcoding Kit (SQK-RPB004) according to the manufacturer’s protocol. DNA was sequenced on an ONT GridION™ Mk1 sequencer using a Min106D flowcell (R 9.4.1). The following settings were set for sequencing: high-accuracy basecalling, minimum q-score of 9, barcoding, and barcode trimming. Samples were sequenced for up to 72 hours, after which basecalled data was transferred for post-processing and analysis.

### 2.4 Bioinformatics pipeline

Bioinformatic processing was carried out using methodology described previously (Greenman et al., 2024). FASTQ files obtained from sequencing were separated into directories for each respective sample. The following pipeline was used to process the data before bioinformatic analyses: First, FASTQ files for a sample were concatenated together into a single FASTQ file. Next, filtering of reads shorter than 1000 bps was accomplished with Filtlong v. 0.2.1 with –min_length 1000 as the only modified option from the defaults (Wick, 2024). Before further filtering, reads were QC’d with NanoPlot v. 1.41.3 with the following parameters: –fastq –plots dot –N50 -o <output_directory> (De Coster and Rademakers, 2023). Host contaminant reads were removed by aligning reads to the mouse reference genome GRCm39 (GCF_000001635.27) with minimap2 v. 2.24-r1122 using the following parameters: -L -ax map-ont <mouse_reference_genome> <reads> (Li, 2018). The generated alignment file was converted to BAM format with samtools v. 1.16.1 using samtools view -b (Danecek et al., 2021). Samtools view -b -f 4 was then used to identify unaligned reads indicating those reads were not from the host genome. Unaligned reads were converted back to FASTQ format with samtools bam2fq using default parameters. NanoPlot was run again on the further filtered reads using the previously described settings. After filtering, metagenomic data was assembled using metaflye v. 2.8.2-b1689 with the following parameters: –nano-raw <filtered_reads> –meta (Kolmogorov et al., 2020). All other parameters were left to their defaults. Following assembly, the filtered reads were mapped to the assembly using minimap2 with the parameter -ax map-ont used to generate an alignment file in SAM format. Polishing of the assembly using the filtered reads and alignment file was first performed with racon v. 1.4.20 using the following parameters: -m 8 -x -6 -g -8 -w 500 -u (Vaser et al., 2017). After racon, further polishing was performed with medaka v. 1.7.2 using medaka_consesus with default parameters except -m which was given r941_min_hac_g507 to denote the flowcell chemistry and high-accuracy basecalling used for our data (“nanoporetech/medaka,” 2024). The filtered reads, referred to henceforth as microbial reads, and the final, polished assembly were used in downstream analyses.

### 2.5 Taxonomic classification, bacterial relative abundance estimation, and diversity

For taxonomic classification, both reads and assembled contigs were classified using Kraken2 v. 2.1.2 (Wood et al., 2019). A report and output file were both generated for reads and assemblies. The option –use-names was used for analysis of both reads and assemblies. For reads, the options –gzip-compressed and –paired were specified. Bracken v. 2.8 was used to estimate relative abundances of taxa within the metagenomes (Lu et al., 2017). A *kmer* database of 1000mers was first created using bracken-build with the following parameters: -d <kraken2_db> -k 35 -l 1000. Once built, bracken was run on reports generated with kraken2 on filtered reads with the following parameters: -d <bracken_db_folder> -i <kraken2_report> -o <bracken_report> -r 1000 -l <P/G/S> where either P, G, or S is chosen depending on what taxonomic rank is analyzed. To minimize the impact of false positive classifications, a minimum relative abundance threshold of 1e^-4^ (1/10,000 reads) was employed. Relative abundances (fraction_total_reads) reported by Bracken were transformed using a center-log ratio (CLR) transformation before statistical analysis (Aitchison, 1982). The CLR was chosen for transforming data because it is scale-invariant and sufficient for non-sparse datasets (Gloor et al., 2017).The natural logarithm was used in the CLR transformation. Count data reported by Bracken were normalized by relative-log expression (RLE) (Anders and Huber, 2010). Figures were generated using a combination of matplotlib v. 3.7.1, seaborn v. 0.12.2, and statannotations v. 0.5.0 (Hunter, 2007; Waskom, 2021; Charlier et al., 2022). Normalized bacterial counts were used to calculate the Bacillota/Bacteroidetes ratio per sample. Values reported in tables were rounded to 4 decimal places. Simpson’s index of diversity was calculated using the alpha_diversity.py script supplied in the KrakenTools suite v. 1.2 (Lu et al., 2022). Bracken reports were supplied to the script and “Si” for Simpson’s index was provided for the -an argument. Substantial differences in abundance were defined as CLR mean differences being ≥ 1 or ≤ -1. A CLR mean difference of ±1 indicates a 2.7-fold greater abundance in a sample type. The sign (+/-) determined whether the taxon was more abundant in PPA or control samples respectively. Significance was determined using a Mann-Whitney U test (Virtanen et al., 2020). Multiple-testing correction was conducted by applying the Benjamini-Hochberg procedure using Statsmodels v. 0.14 (Benjamini and Hochberg, 1995; Seabold and Perktold, 2010). A p-value of ≤ 0.05 after correction was used as a threshold for determining statistical significance.

### 2.6 Functional annotation and gene relative abundance estimation

Gene annotation and relative abundance estimation were accomplished with a modified version of the protocol described by Maranga et al (Maranga et al., 2023). First, all assemblies had contigs shorter than 500 bps removed using SeqKit v. 2.5.1 (Shen et al., 2016). The filtered assemblies were then concatenated into a pan-metagenome. Pprodigal v. 1.0.1, a parallelized version of Prodigal v. 2.6.3, was used to identify open reading frames (ORFs) with the following parameters: -d <nucleotide_file> -f gff -i <pan-metagenome> -o <output_file> -T 24 -p meta -C 10000 (Hyatt et al., 2012; Jaenicke, 2024). The nucleotide file generated was then filtered with python to remove all partial genes. Next, CD-HIT v. 4.8.1 was used to cluster genes with the following parameters: cd-hit-est -i <filtered_nucleotide_file> -o <non-redundant_gene_catalog> -c 0.95 -s 0.85 -aS 0.9 -n 10 -d 256 -M 350000 -T 24 -l 100 -g 1 (Fu et al., 2012). The resulting non-redundant gene catalog was used for gene abundance estimation and annotation. Relative gene abundance was estimated with KMA v. 1.4.9 (Clausen et al., 2018). First, an index file was made using KMA index with the following parameters: -i <non-redundant_gene_catalog> -o <index_db>. Then, using the index generated along with each sample’s microbial reads described in the bioinformatics pipeline section, KMA was run using the following parameters: -i <microbial_reads> -o <output_directory> -t_db <index_db_path> -bcNano -bc 0.7 -ef -t 24. Gene counts were then normalized using CLR for principal component analysis (PCA) using Sci-kit learn’s PCA class (Pedregosa et al., 2011). Annotation of predicted genes was performed using eggNOG v. 2.1.12’s emapper.py script and eggNOG database version 5.0.2 on the non-redundant gene catalog with the following parameters: –itype CDS –cpu 24 -i <non-redundant_gene_catalog> –data_dir <eggNOG_db_directory> –go_evidence non-electronic –output <output_file_prefix> –output_dir <output_directory> –target_orthologs all –seed_ortholog_evalue 0.001 –seed_ortholog_score 60 –query_cover 20 –subject_cover 0 –translate –override –temp_dir <temp_file_directory> (Cantalapiedra et al., 2021). KMA results were filtered to select for genes that had sufficient Template_Coverage and Template_Identity (≥ 90%) and prevalence (Depth ≥ 3). KMA Depth results were transformed using CLR as described earlier. Results from KMA were then matched using the contig source for each gene to the contig ID in the functional annotation and taxonomic classification results. As with taxa, substantial differences in gene abundance were defined by genes possessing a CLR mean difference ≥ 1 or ≤ -1, with the sign (+/-) determining a gene’s abundance being greater in PPA or control samples respectively.

Comparison of gene pathway abundance was performed by first clustering genes according to their unique Kyoto Encyclopedia of Genes and Genomes (KEGG) orthology (KO) ID assigned by eggNOG. Genes without a KO or genes with multiple KOs were removed prior to analysis. The average abundance of each KO in a sample was then calculated before statistical analysis. PPA metabolism genes were defined as any genes assigned the string ko00640 in the KEGG_Pathway column, indicating its role in propanoate metabolism according to KEGG. Identification of genes associated with PPA production are listed in **Supplementary Table 1** (Reichardt et al., 2014; Yang et al., 2017). Permutation testing was performed to identify PPA metabolism and production genes significantly more abundant in each sample type. One thousand permutations were performed for each gene analyzed. A p-value of 0.05 was used as a threshold for determining statistical significance. Functional annotations were assigned to individual genes within a cluster according to the annotation for the representative gene of that cluster. Identification of taxa associated with PPA metabolism and/or PPA production was accomplished by matching contig IDs from Kraken2’s output file to identical contig IDs preserved during functional annotation with eggNOG. Significance testing was performed using a Mann-Whitney U test as described previously. Multiple-testing correction was performed using the Benjamini-Hochberg procedure. A p-value of ≤ 0.05 was used as a threshold for determining statistical significance.

## 3 Results

### 3.1 Altered microbial composition in PPA samples

**Figure 1:**
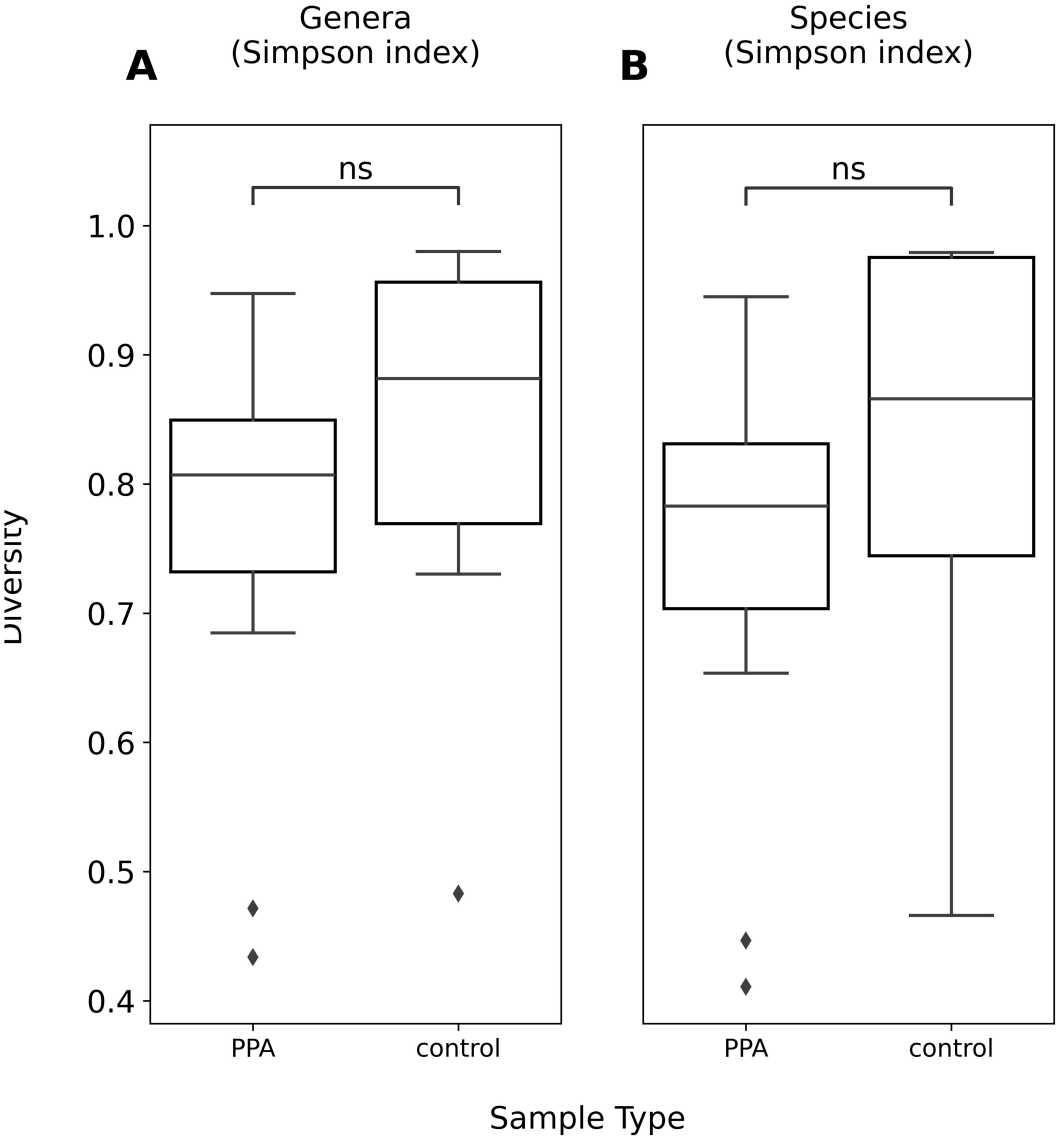
Alpha diversity of genera and species compositions of murine gut microbiomes. Boxplots depict Simpson’s index of diversity of genera **(A)** and species **(B)** in PPA and Control samples. Significance was determined using a Mann–Whitney U test with correction for multiple tests by Benjamini-Hochberg procedure. ns, non-significant p–value (p > 0.05).

**Figure 2:**
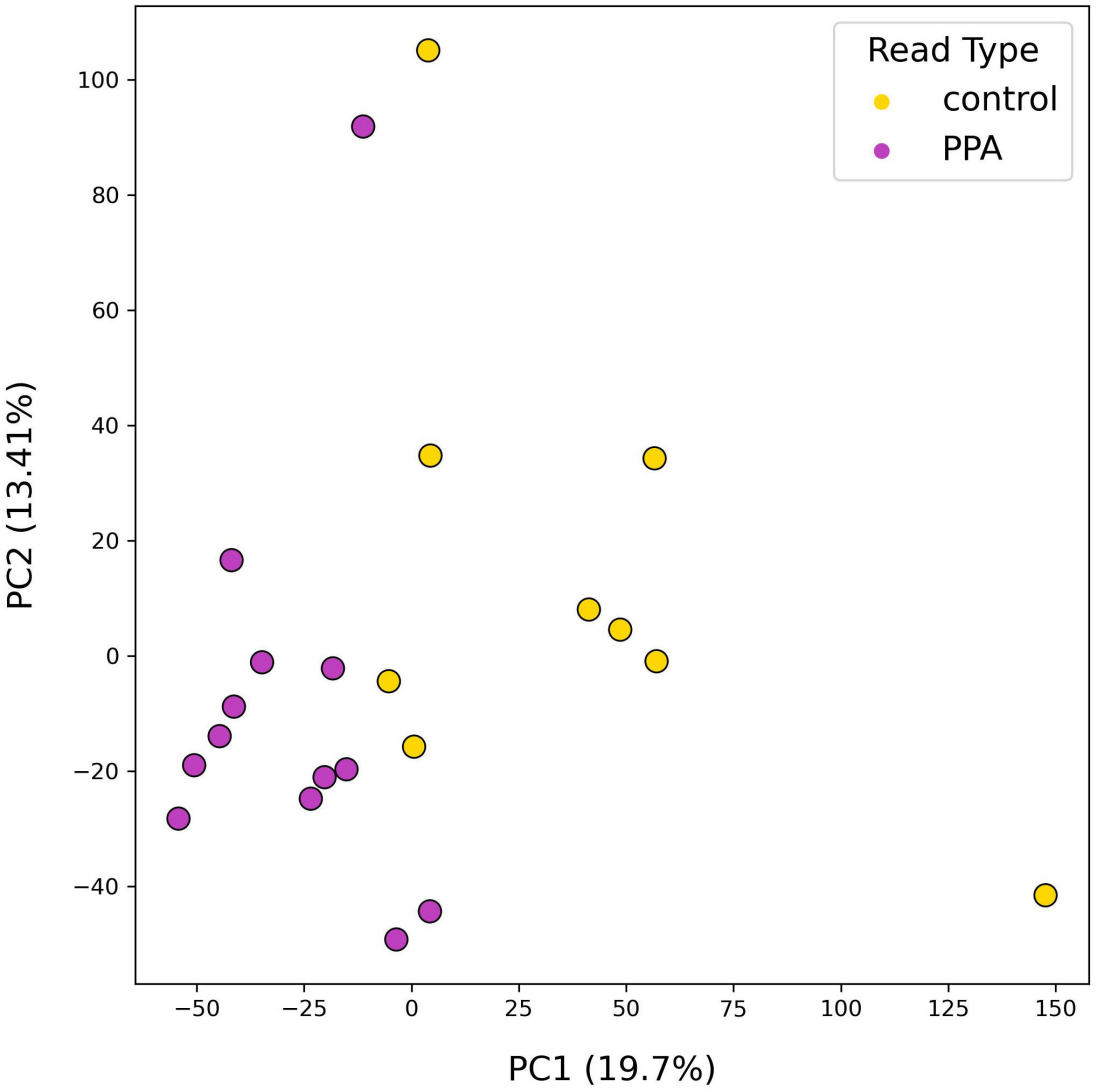
PCA results of species-level mouse gut microbiome compositions. PCA plot depicts the distributions of samples according to their top two principal components. Colors denote the sample type, with PPA-exposed mice in purple and control mice in yellow. Principal components 1 and 2 are listed with their explained variance ratios as percentages on the x and y-axis respectively.

The diversity of murine gut microbiomes was assessed using Simpson’s diversity index. Control and PPA samples were not significantly different in terms of genera and species diversity (Genera p-value: 0.18, Species p-value: 0.16) (**Figure 1**). Next, microbial compositions were compared using PCA. **Figure 2** shows clustering of samples according to their sample type, indicating a difference in what species make up the microbiome of PPA and control samples. At the genus level, this clustering was not as strong, suggesting PPA affects specific bacteria (**Supplementary Figure 1**).

Using RLE transformed count data, a significant decrease in the median Bacillota/Bacteroidota ratio between control and PPA samples was observed (Control: 9.66, PPA: 3.02; p-value = 0.0011). This difference resulted from PPA mice possessing a greater abundance of Bacteroidota relative to the control, although not significantly different (Control mean CLR: 5.51, PPA mean CLR: 6.62; p-value = 0.054) while Bacillota abundance was similar (Control mean CLR: 7.76, PPA mean CLR: 7.60; p-value = 0.18).

**Figure 3:**
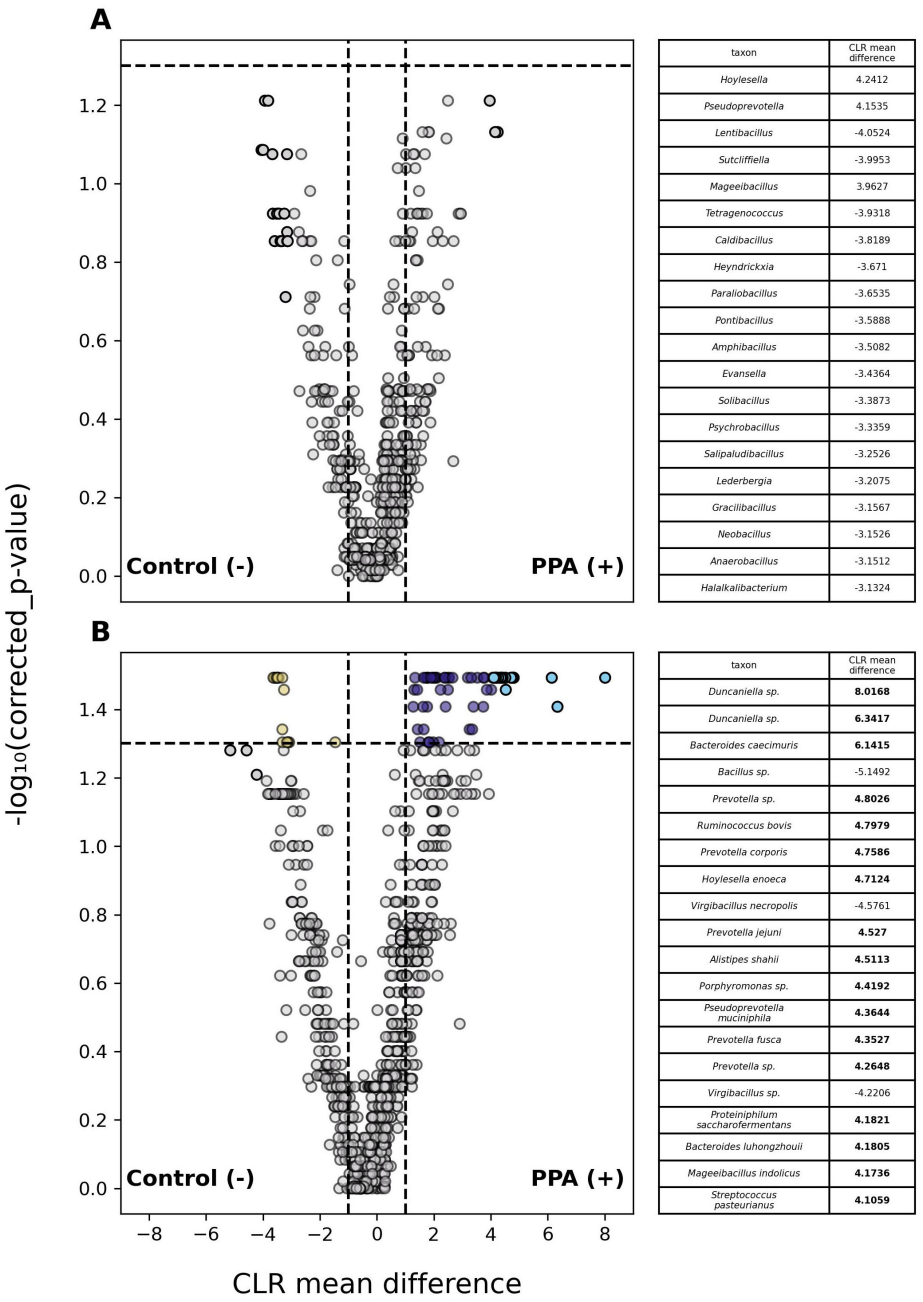
Differential abundance of taxa in PPA and Control murine gut microbiomes. Volcano plots depict differences in the abundances of genera **(A)** or species **(B)** between PPA and control samples. Gray points indicate taxa whose abundances between taxa did not significantly differ. Colored points indicate a significant difference in abundance (p–value ≤ 0.05). The top 20 taxa with the greatest difference in abundance between sample types are red and light blue for control and PPA samples respectively. Yellow and purple points were at least 2.7 times more abundant in control or PPA samples. Black points indicate taxa with a significant difference in abundance, whose CLR mean difference was between -1 and 1. P–values were calculated using a Mann–Whitney U test with correction for multiple tests by Benjamini-Hochberg procedure. Bold CLR mean difference values indicate a significant difference in abundance.

Analysis of abundances in classified members of the gut microbiome revealed 1 phylum and 77 species that differed significantly between PPA and control samples (**Supplementary Table 2**). Fifty-nine species were substantially more abundant in PPA samples, whereas only 16 species were more abundant in control samples (**Figure 3**).

### 3.2 Functional annotation of murine gut metagenomes

**Figure 4:**
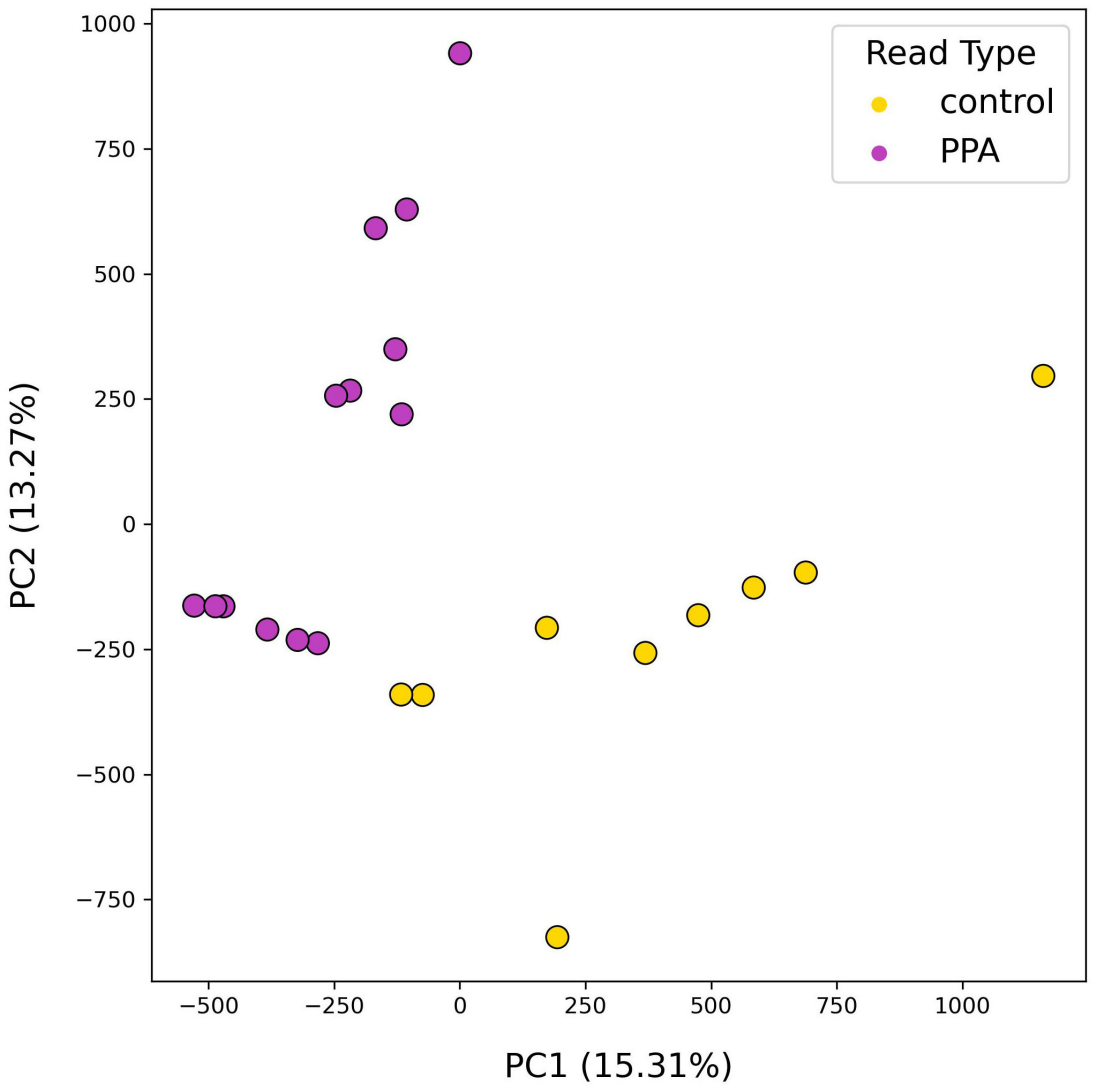
PCA results using functional profiles of mouse gut microbiomes. PCA plot depicts the distributions of samples according to their top two principal components. Colors denote the sample type, with PPA-exposed mice in purple and control mice in yellow. Principal components 1 and 2 are listed with their explained variance ratios as percentages on the x– and y–axis respectively.

Following analysis of gut microbial compositions, functional annotation of microbiomes was performed. A total of 378,355 unique genes were identified across all samples after filtering out low quality genes. The transformed abundances of these genes were used in PCA, revealing strong clustering of sample type based on their functional profiles (**Figure 4**).

**Figure 5:**
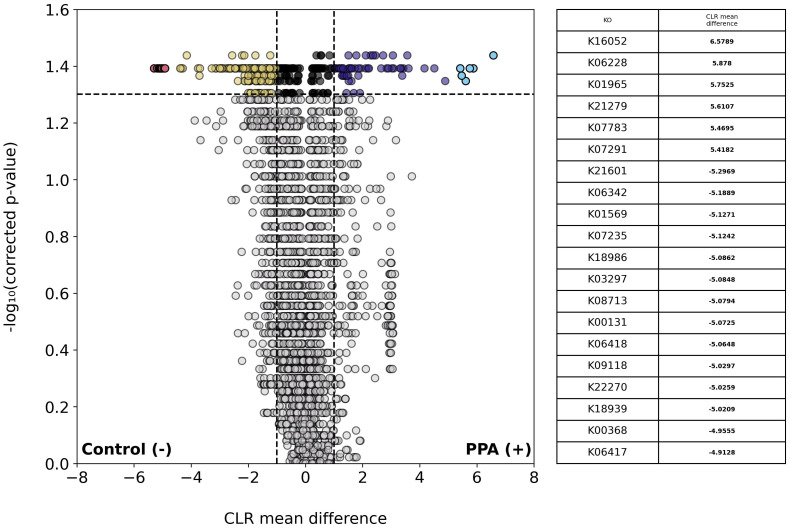
Differential abundance of KOs in PPA and control murine gut microbiomes. Volcano plot depicts differences in the abundance of functional groups (KOs). Gray points indicate KOs whose abundances did not significantly differ between sample types (p–value > 0.05). Colored points indicate a significant difference in abundance (p–value ≤ 0.05). The top 20 KOs with the greatest difference in abundance between sample types are red and light blue for control or PPA samples respectively. Yellow and purple points are KOs at least 2.7 times more abundant in control or PPA samples, respectively. Black points indicate KOs with a significant difference in abundance, whose CLR mean difference was between -1 and 1. P-values were calculated using a Mann–Whitney U test with correction for multiple tests by Benjamini-Hochberg procedure. NaN indicates a KO was not a part of a pathway in KEGG. Bold CLR mean difference values indicate a significant difference in abundance. For details on pathways the KOs listed belong to, see **Supplementary Table 3**.

We next examined the abundances of KEGG KOs across sample types. A total of 3,648 unique KOs were identified, of which 196 were significantly more abundant in control samples and 106 were more abundant in PPA samples (**Figure 5**). One-hundred and forty-five genes in control samples and 61 genes in PPA samples showed a substantial difference in abundance. Pathways associated with lipid metabolism and amino sugar metabolism were significantly more abundant in PPA samples (**Supplementary Table 3**). Control samples showed significantly more abundant pathways associated with nitrogen metabolism and sulfur relay systems (**Supplementary Table 3**). PPA samples had a significantly higher abundance of genes associated with amino sugar/nucleotide sugar metabolism (ko:K21279) and Inositol phosphate metabolism (ko:K07291) (**Figure 5**). Control samples possessed significantly more genes associated with benzoate metabolism (ko:K22270), nitrogen metabolism (ko:K00368), and glycolysis/gluconeogenesis (ko:K00131) (**Figure 5**).

**Figure 6:**
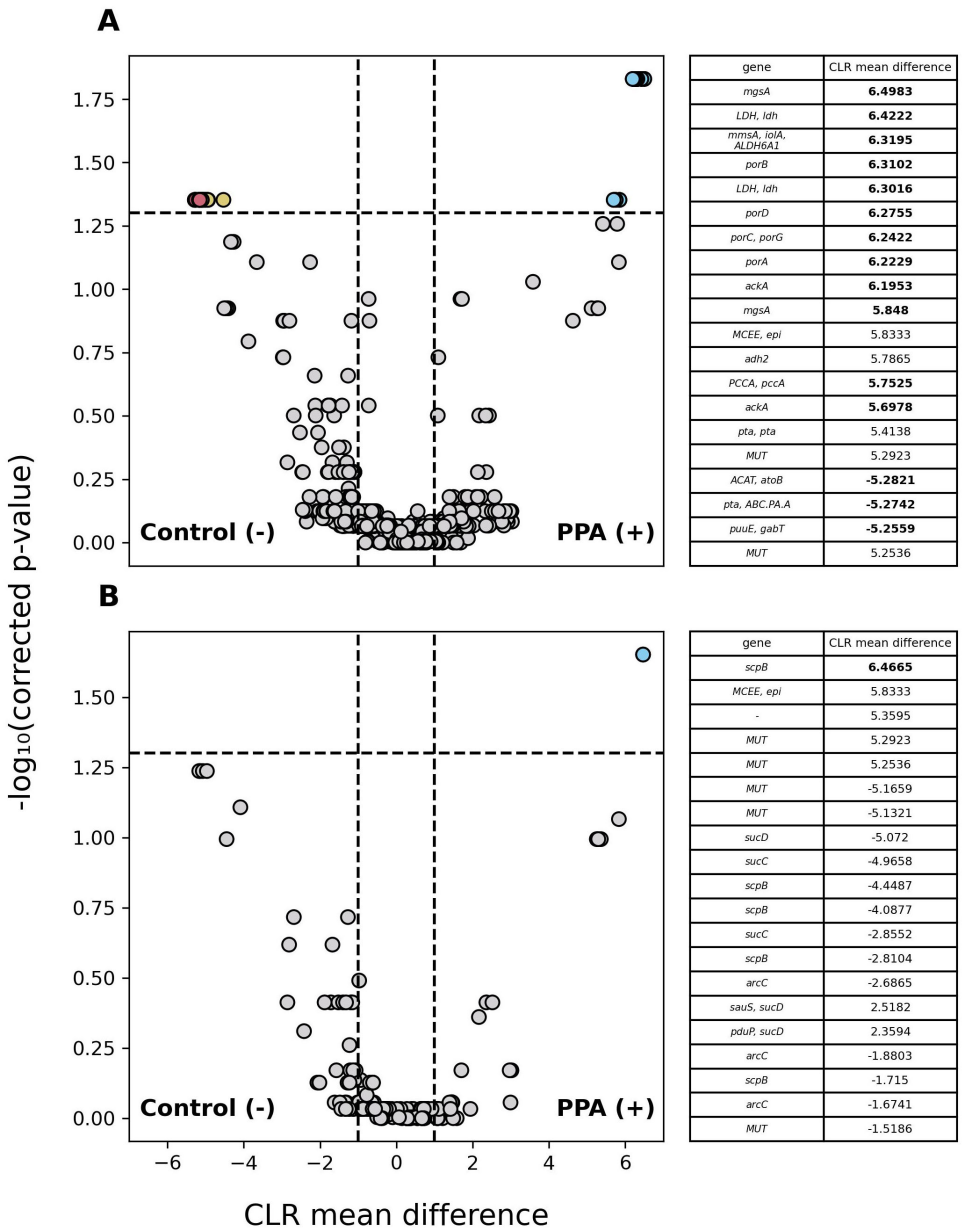
Differential abundance of PPA-associated genes in murine gut microbiomes. Volcano plots depict differences in the abundance of genes associated with PPA metabolism **(A)** and PPA production **(B)**. Gray points indicate genes whose abundance did not significantly differ between sample types (p–value > 0.05). Colored points indicate a significant difference in abundance (p–value ≤ 0.05). The 20 genes with the greatest difference in abundance are red and light blue for control or PPA samples respectively. Yellow and Purple points were at least 2.7 times more abundant in control or PPA samples. Black points indicate genes with a significant difference in abundance, whose CLR mean difference was between -1 and 1. P-values were calculated using a Mann–Whitney U test with correction for multiple tests by Benjamini-Hochberg procedure. Genes correspond to the representative genes in the non-redundant gene catalog. Gene names consist of the symbols for a gene’s KO according to KEGG. Bold CLR mean difference values indicate a significant difference in abundance. A dash (-) indicates no symbol was available for a gene according to KEGG.

Among annotated genes, 1,601 significantly differed in abundance (p ≤ 0.05) between sample types with genes being at least 2.7 times more abundant in either. Of those genes, 4 were more abundant in control samples and 1,597 were more abundant in PPA samples. Because PPA has antimicrobial properties, we examined the abundance of PPA metabolism and production genes between sample types. Out of 1332 genes associated with PPA metabolism, 27 were significantly more abundant in control samples and 12 were more abundant in PPA samples. Out of 223 genes associated with PPA production, 1 gene was significantly more abundant in PPA samples. **Figure 6A** further demonstrates the higher prevalence of PPA metabolism-associated genes with significantly higher abundances of a substantial effect size being present in the control samples, while 6B highlights the singular gene with a significantly greater abundance observed in PPA samples.

### 3.3 Taxa associated with PPA metabolism and/or production

Taxa possessing genes associated with PPA metabolism and/or production were identified by matching a contig’s taxonomic identity to the contig ID for a given gene. At the genus level, 130 genera were found to possess genes associated with PPA metabolism and 61 possessed genes associated with PPA production (**Supplementary Table 4**). No genera however showed a significant difference in abundance (p > 0.05).

At the species level, 144 were found with genes associated with PPA metabolism and 68 bacteria were found with genes associated with PPA production (**Supplementary Table 5**). Within the PPA metabolizers, 8 bacteria showed a significant increase in abundance between sample types with all of them having a sizable effect change (**Supplementary Table 6**). All of the PPA metabolizers with a substantial difference in abundance identified were more abundant in PPA samples. Species-level classification revealed members whose genera did not significantly differ between sample types, including several *Bacteroides* and *Ruminococcus* species, as well as *Duncaniella dubosii, Muribaculum intestinale,Monoglobus pectinilyticus*, and *Sodaliphilus pleomorphus*. Among PPA producers, the abundance of 4 were significantly different between sample types. Those with a notable difference in abundance included *Bacteroides nordii, Duncaniella dubosii, Muribaculum intestinale*, and *Ruminococcus bovis*.

## 4 Discussion

In this study we investigated the effects of dietary PPA exposure on the murine gut microbiome. PPA may elicit a number of responses among bacteria since it is produced by select species, used as a nutrient source by others, or have an antimicrobial effect. As a result, its addition to the gut environment through dietary supplementation likely has a differential effect depending on tolerance, susceptibility, and ability to use it as a nutrient. Sensitive species are likely removed and replaced by those with higher PPA-tolerance or ability to use it as a food source, resulting in changes in the composition of the gut microbiome. Our results identified significant differences in microbial composition with no effect on overall microbial diversity. The greatest effect was observed at the species level, with over 70 taxa differing significantly in abundances between PPA and control samples (**Supplementary Table 2**). Further assessment of the composition of the PPA exposed sample showed greater heterogeneity in microbial species compared to the unexposed sample, suggesting that PPA may augment bacterial growth characteristics and limit the taxa that survive in a PPA-rich environment. As a result, PPA may induce alterations selectively instead of producing widespread disruptions to gut microbiome diversity.

Food preservatives such as PPA have previously been shown to alter the abundances of gut microbiome constituents without affecting overall diversity (Nagpal et al., 2021). Here, we observed the most notable difference among species of the *Bacteroides*, belonging to the phylum Bacteroidota (previously Bacteroidetes), which were significantly more abundant in PPA exposed mice. Increased *Bacteroides* has been linked with increased mucus degradation, which may increase risk of infection and promote inflammation (Cornick et al., 2015; Desai et al., 2016; Penzol et al., 2019). In one study, newborn male mice treated with *Bacteroides fragilis* were found to exhibit ASD-like social behavior (Carmel et al., 2023), and other research has demonstrated that species of *Bacteroides* can alter immune activity, resulting in autoimmune inflammatory cardiomyopathy (Gil-Cruz et al., 2019). Species belonging to the genera *Ruminococcus, Prevotella*, and *Parabacteroides* were also significantly higher in abundance in the PPA exposed mice (Coretti et al., 2018). Certain *Ruminococcus* species have been linked to disorders like Chron’s Disease through production of a pro-inflammatory cytokines (Henke et al., 2019), and *Prevotella* species, such as *Prevotella copri*, have been associated with metabolic-related conditions like hypertension and insulin sensitivity (Pedersen et al., 2016; Li et al., 2017). Last, we found that the ratio of Bacillota (previously Firmicutes) to Bacteroidota was significantly lower in PPA exposed mice compared to control mice due to higher overall abundance of Bacteroidota species. This ratio has previously been shown to be a significant indicator of gut homeostasis, and disruptions in this ratio have been linked to several disease states (Turpin et al., 2016; Takezawa et al., 2021; An et al., 2023), including inflammatory bowel disease (Stojanov et al., 2020). Taken together, species belonging to phylum Bacteroidota appear to be the most significantly impacted by increased dietary PPA. This may result from higher tolerance to PPA or the ability to use PPA as an energy source, which has been shown true for at least one member species, *Hoylesella enocea* (Hitch et al., 2022). Alternatively, maternal PPA exposure may augment prenatal development, allowing for the guts of progeny mice to be more easily colonized by *Bacteroides*; however, our study design did not allow for this assessment.

Assessment of metagenome content found significant differences in abundances of genes associated with PPA metabolism and production, with PPA exposed mice possessing a greater abundance of PPA production genes and unexposed mice PAA metabolism (**Figure 6**). These results suggest that the effect of PPA on microbial composition may not solely be due to its utilization, otherwise PPA metabolism-associated gene abundance should show greater abundance in the gut microbiomes of PPA exposed mice. One explanation is that PPA mediates bacterial abundance mainly through its antimicrobial effects as opposed to bacteria utilizing it as a nutrient. Previous work has demonstrated that, in the case of *Salmonella Typhimurium*, PPA inhibited growth in a dose-dependent manner (Jacobson et al., 2018). Exposure to higher levels of PPA may select for bacteria that are resistant to its antimicrobial properties while not necessarily being able to metabolize or produce it. For example, several *Parabacteroides* species displayed significantly greater abundance in PPA samples, however no genes linked to PPA metabolism or production were found to be associated with them (**Supplementary Tables 2, 4, 5**). Furthermore, production of PPA as a fermentation byproduct occurs across a wide range of bacteria (Gonzalez-Garcia et al., 2017). Greater bacterial diversity may account for the greater abundance of PPA metabolism-associated genes in control samples (Averina et al., 2020). Also, only 27/1332 (2.14%) genes were predicted to be ones associated with only PPA metabolism. Many genes associated with PPA metabolism are also a part of other metabolic pathways. This provides further evidence for the greater abundance of PPA metabolism-associated genes in control samples; these genes may be functioning in pathways that do not result in PPA being used or produced as a byproduct. Here, only one gene associated with PPA production showed a significant difference in abundance between sample types. Unlike PPA metabolism-associated genes, marker genes for PPA production were chosen because they directly participate in bacterial PPA production pathways. All species that showed significant increased abundances and the ability to produce PPA were found in PPA exposed mice. This supports the prediction that PPA selects for PPA producers, thus an increased capacity for PPA production is predicted. Gene abundance, however, does not necessarily correlate to gene expression; therefore, it is possible that while a larger abundance of PPA metabolism-associated genes were in control samples, the rates of expression may vary (Shi et al., 2014). Research into PPA production-associated gene expression is required to support an association between PPA-production gene abundance and PPA production.

Several differences were observed when the results of functional annotation for PPA and control metagenomes. PCA analysis of gene content resulted in discrete clusters forming between PPA and control samples (**Figure 5**). Intra-sample clustering revealed that control gene content was more diverse, whereas PPA samples clustered near each other. Clustering by gene content was comparable to clustering by species composition. Differences in the abundance of metabolic pathways thus coincide with changes in the abundance of specific species and strains therein. In PPA samples, two pathways that saw significantly higher abundance were those associated with amino sugar/nucleotide sugar metabolism (ko:K21279) and multiple lipid-metabolism pathways (ko:K00647, ko:K03801; **Supplementary Table 3**). The gene associated with ko:K21279 is known to be associated with *Bacteroides*, one of the genera with species that were significantly more abundant in PPA samples. This enzyme may enable immune avoidance through expression of capsular polysaccharides (Wang et al., 2008). This could contribute to the observed increased abundance of *Bacteroides* in PPA-exposed mice. This complements the finding that increased fatty acid synthesis was observed in PPA microbiomes. Bacteria utilize the FASII pathway ko:K00647 (*fabB*) to produce fatty acids that can impact host metabolic pathways (Yao and Rock, 2015; Johnson et al., 2020), and altered lipid metabolism may play a role in neurodevelopment (Yu et al., 2020). Another pathway showing increased abundance in PPA samples was steroid hormone biosynthesis (ko:K12343). Growing evidence shows an interconnected relationship between the gut microbiota’s ability to influence and be influenced by hormone levels, thus increased steroid levels may have downstream health effects (Tetel et al., 2018).

This study is not without limitations or considerations. One important distinction is that animals were not physiologically evaluated. Therefore, association of microbiome changes with any disease cannot be concluded directly. Another consideration is that mice in this study were kept on the same diet as the mothers. Future work may include identifying if switching from a PPA-rich to a PPA-free diet ameliorates its effects on the microbiome. One limitation our study shares with many others is the limited sample size. Although valid inferences can be drawn, larger sample sizes provide more statistical power when analyzing results. We also caution on drawing conclusions related to the association between changes in the gut microbiome and any disease (Yap et al., 2021). Confounding factors, including age, sex, and diet can all have significant effects on microbial composition. These factors may explain inconsistencies observed in the literature regarding gut microbiome association with complex conditions (Johnson et al., 2019; Lagod and Naser, 2023). For example, members of the genus *Bacteroides* have been shown to have either an increased or decreased abundance in animals and individuals with ASD (Angelis et al., 2013; Kushak et al., 2017).Similarly, studies of gut compositions in individuals with inflammatory bowel diseases have reported both increased and decreased abundance for the same taxa (Walters et al., 2014; Forbes et al., 2018; Upadhyay et al., 2023). To limit the influence of sex-related bias, we attempted to have equal representation of sexes such that differences were likely the result of diet. One challenge in functional annotation was the removal of redundant gene sequences. Our gene clustering approach required 95% sequence identity with an 85% length similarity, and 90% alignment coverage to mitigate spurious clustering. However, in some instances, we observed COGs with the same annotation (e.g., *MUT*) (**Figure 6**). Further investigation is needed to determine if these are distinct orthologs associated with specific genera or a limitation in the gene cluster approach. Another limitation with functional annotation dealt with possible misclassifications; the bacterial gene *mmdA* is a known enzyme associated with the PPA synthesis, yet KEGG does not associate it with the propanoate metabolism pathway. Instead, orthologs *scpB* and *mmcD* are. Numerous genes not having assigned KOs could result in PPA-associated genes being unaccounted for when estimating gene abundance. Future work would benefit from analyzing the meta-transcriptome, which may offer greater insight into the functional profiles of the gut microbiota and tie gene expression to potential downstream effects. For work related to specific neurodevelopmental conditions or inflammatory bowel diseases, physiological and behavioral evaluation of animals should be performed to associate changes in microbial composition with said condition. Complementary research of gut microbiome transplantation into germ-free mice to observe whether the microbiome is a factor or feature of a condition would also be beneficial.

In conclusion, we demonstrated the role of dietary PPA as an factor that contributes to variation in microbial composition of the gut microbiome. An FDA-approved preservative, PPA’s prevalence in various food products offers routes for extended exposure that can lead to disruption of normal gut flora. We identified abundance changes to several bacterial species, demonstrating PPA’s ability to affect the composition of the gut microbiome. An altered microbiota can translate to altered levels of certain metabolic pathways, potentially contributing to physiological changes linked to host health. Future studies are needed to examine whether alterations to microbiome composition from dietary PPA induces dysbiosis or the development of other conditions. This study provides a foundation toward future investigations into how PPA’s effect on gut composition affects one’s health.

## Data Availability

The datasets presented in this study can be found in online repositories. The names of the repository/repositories and accession number(s) can be found below: https://www.ncbi.nlm.nih.gov/, PRJNA1092431.
