# Supplementary material for: Impact of propionic acid-rich diets on microbial composition of the murine gut microbiome
Source: Front Microbiomes. 2024 Oct 22;3:1451735. doi: 10.3389/frmbi.2024.1451735 (PMC12993523; doi:10.3389/frmbi.2024.1451735)
Supplement: Supplementary file 1 [file DataSheet1.docx]

Supplementary Material

# Supplementary Figures and Tables

For more information on Supplementary Material and for details on the different file types accepted, please see [here](https://www.frontiersin.org/guidelines/author-guidelines#supplementary-material).

## Supplementary Figures


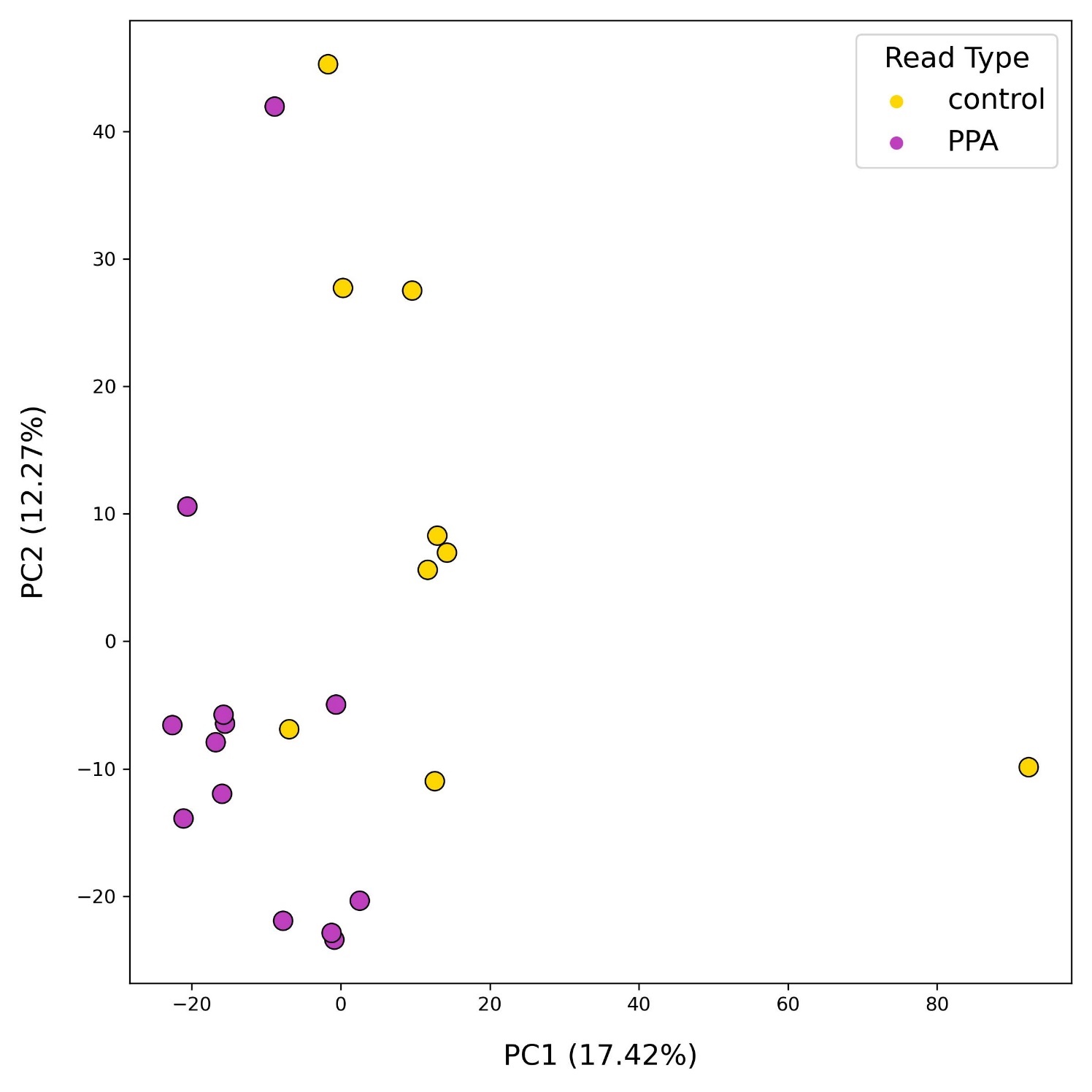


Supplemental Figure 1. PCA results of genus-level mouse gut microbiome compositions. PCA plot depicts the distributions of samples according to their top two principal components. Colors denote the sample type, with PPA-exposed mice in purple and control mice in yellow. Principal components 1 and 2 are listed with their explained variance ratios as percentages on the x– and y–axis respectively.

## Supplementary Tables

Supplementary Table 1. PPA Production Genes

| Gene | Enzyme | Associated PPA production pathway |
| --- | --- | --- |
| *sucC* | succinyl-CoA synthetase² | P1 |
| *sucD* | succinyl-CoA synthetase² | P1 |
| *LSC1* | succinyl-CoA synthetase² | P1 |
| *LSC2* | succinyl-CoA synthetase² | P1 |
| *mutA* | methylmalonyl-CoA mutase² | P1 |
| *mutB* | methylmalonyl-CoA mutase² | P1 |
| *mcee* | methylmalonyl-CoA/ethylmalonyl-CoA epimerase² | P1 |
| *mmdA* | methylmalonyl-CoA decarboxylase¹,² | P1 |
| *scpB* | methylmalonyl-CoA decarboxylase³ | P1 |
| *pct* | propionate CoA-transferase² | P2 |
| *lcdA* | lactoyl-CoA dehydratase¹,² | P2 |
| *arcC* | acrylyl-CoA reductase² | P2 |
| *pduC* | propanediol dehydratase (large subunit)² | P3 |
| *pduD* | propanediol dehydratase (medium subunit)² | P3 |
| *pduE* | propanediol dehydratase (small subunit)² | P3 |
| *pduP* | propanediol dehydrogenase¹,² | P3 |
| *pduQ* | propanediol dehydratase¹ | P3 |

^1^Referenced by (Reichardt et al., 2014)

^2^Referenced by (Yang et al., 2017)

^3^Shared orthology with present gene (*mmdA*)

^4^P1: Succinate pathway, P2: Lactate pathway, P3: 1,2–propanediol pathway

Supplementary Table 2. Taxa displaying significantly different abundances between PPA and control samples

| taxa | taxa level¹ | statistic² | p_value³ | control mean relative abundance | PPA mean relative abundance | CLR mean difference | abundance in PPA⁴ |
| --- | --- | --- | --- | --- | --- | --- | --- |
| Chlorobiota | P | 8 | 0.0264 | -1.2365 | 0.3548 | 1.5913 | ↗ |
| *Aeromonas veronii* | S | 12 | 0.0490 | 1.4755 | 3.6594 | 2.1839 | ↗ |
| *Alistipes onderdonkii* | S | 10 | 0.0377 | 2.7843 | 4.5508 | 1.7665 | ↗ |
| *Alistipes senegalensis* | S | 10 | 0.0377 | -1.3647 | 2.3721 | 3.7368 | ↗ |
| *Alistipes shahii* | S | 3 | 0.0306 | -0.2054 | 4.3059 | 4.5113 | ↗ |
| *Alkalihalobacillus sp. LMS39* | S | 109 | 0.0306 | 2.4075 | -1.096 | -3.5035 | ↘ |
| *Bacillus sp. Y1* | S | 109 | 0.0306 | 2.446 | -1.1211 | -3.5671 | ↘ |
| *Bacteroides caecimuris* | S | 4 | 0.0306 | 0.1038 | 6.2453 | 6.1415 | ↗ |
| *Bacteroides faecis* | S | 6 | 0.0306 | 3.0724 | 4.8487 | 1.7763 | ↗ |
| *Bacteroides faecium* | S | 6 | 0.0306 | 2.5944 | 4.3652 | 1.7708 | ↗ |
| *Bacteroides fragilis* | S | 11 | 0.0456 | 4.682 | 6.1121 | 1.4301 | ↗ |
| *Bacteroides helcogenes* | S | 12 | 0.0490 | 2.6866 | 4.2028 | 1.5162 | ↗ |
| *Bacteroides intestinalis* | S | 10 | 0.0377 | -0.2753 | 3.1153 | 3.3906 | ↗ |
| *Bacteroides luhongzhouii* | S | 8 | 0.0306 | -1.4201 | 2.7604 | 4.1805 | ↗ |
| *Bacteroides nordii* | S | 4 | 0.0306 | 2.8439 | 6.0355 | 3.1916 | ↗ |
| *Bacteroides ovatus* | S | 5 | 0.0306 | 0.9192 | 4.6925 | 3.7733 | ↗ |
| *Bacteroides sp. M10* | S | 9 | 0.0360 | -1.9907 | 2.0263 | 4.017 | ↗ |
| *Bacteroides thetaiotaomicron* | S | 6 | 0.0306 | 3.7271 | 5.0681 | 1.341 | ↗ |
| *Bacteroides zhangwenhongii* | S | 12 | 0.0490 | 2.3338 | 4.3102 | 1.9764 | ↗ |
| *Butyricimonas virosa* | S | 8 | 0.0306 | -1.3818 | 2.6835 | 4.0653 | ↗ |
| *Caldibacillus thermoamylovorans* | S | 113 | 0.0306 | 2.1125 | -1.4955 | -3.608 | ↘ |
| *Caproicibacterium amylolyticum* | S | 10 | 0.0377 | 2.887 | 4.1636 | 1.2766 | ↗ |
| *Caproicibacterium sp. BJN0003* | S | 9 | 0.0360 | 2.5584 | 3.8703 | 1.3119 | ↗ |
| *Cytobacillus firmus* | S | 110 | 0.0306 | 2.323 | -1.1272 | -3.4502 | ↘ |
| *Duncaniella dubosii* | S | 7 | 0.0306 | 5.8039 | 7.5896 | 1.7857 | ↗ |
| *Duncaniella freteri* | S | 8 | 0.0306 | 1.7012 | 4.1942 | 2.493 | ↗ |
| *Duncaniella sp. B8* | S | 10 | 0.0377 | 0.6614 | 7.0031 | 6.3417 | ↗ |
| *Duncaniella sp. C9* | S | 1 | 0.0306 | -0.9803 | 7.0365 | 8.0168 | ↗ |
| *Epilithonimonas vandammei* | S | 11 | 0.0456 | -1.9907 | 1.2636 | 3.2543 | ↗ |
| *Gracilibacillus sp. SSPM10-3* | S | 109 | 0.0306 | 2.3746 | -1.1339 | -3.5085 | ↘ |
| *Gracilibacillus sp. SSWR10-1* | S | 113 | 0.0306 | 2.1414 | -1.4955 | -3.6369 | ↘ |
| *Hoylesella enoeca* | S | 6 | 0.0306 | -1.3367 | 3.3757 | 4.7124 | ↗ |
| *Lysinibacillus sp. Ag94* | S | 106 | 0.0456 | 2.2117 | -1.1101 | -3.3218 | ↘ |
| *Mageeibacillus indolicus* | S | 4 | 0.0306 | -0.825 | 3.3486 | 4.1736 | ↗ |
| *Metabacillus sp. B2-18* | S | 109 | 0.0306 | 2.3925 | -1.1272 | -3.5197 | ↘ |
| *Monoglobus pectinilyticus* | S | 11 | 0.0456 | 2.0833 | 3.7277 | 1.6444 | ↗ |
| *Muribaculum intestinale* | S | 7 | 0.0306 | 5.6228 | 7.5802 | 1.9574 | ↗ |
| *Niallia circulans* | S | 105 | 0.0490 | 3.3414 | 0.0242 | -3.3172 | ↘ |
| *Odoribacter splanchnicus* | S | 6 | 0.0306 | -0.2973 | 3.2292 | 3.5265 | ↗ |
| *Parabacteroides distasonis* | S | 4 | 0.0306 | 2.0002 | 4.5436 | 2.5434 | ↗ |
| *Parabacteroides faecis* | S | 7 | 0.0306 | 2.0551 | 4.1651 | 2.11 | ↗ |
| *Parabacteroides goldsteinii* | S | 10 | 0.0377 | 2.937 | 4.5597 | 1.6227 | ↗ |
| *Parageobacillus toebii* | S | 105 | 0.0490 | 1.5856 | -1.4955 | -3.0811 | ↘ |
| *Petrimonas mucosa* | S | 5 | 0.0306 | 1.9905 | 4.0348 | 2.0443 | ↗ |
| *Phocaeicola coprophilus* | S | 9 | 0.0360 | 1.5149 | 4.0137 | 2.4988 | ↗ |
| *Phocaeicola dorei* | S | 6 | 0.0306 | 2.7549 | 4.4631 | 1.7082 | ↗ |
| *Phocaeicola vulgatus* | S | 8 | 0.0306 | 2.907 | 4.5501 | 1.6431 | ↗ |
| *Porphyromonas cangingivalis* | S | 11 | 0.0456 | -0.3187 | 3.0294 | 3.3481 | ↗ |
| *Porphyromonas gingivalis* | S | 9 | 0.0360 | -0.8311 | 3.0273 | 3.8584 | ↗ |
| *Porphyromonas sp. oral taxon 275* | S | 8 | 0.0306 | -1.3915 | 3.0278 | 4.4193 | ↗ |
| *Prevotella bryantii* | S | 6 | 0.0306 | 0.2428 | 3.5559 | 3.3131 | ↗ |
| *Prevotella copri* | S | 9 | 0.0360 | 2.1084 | 4.3442 | 2.2358 | ↗ |
| *Prevotella corporis* | S | 6 | 0.0306 | -1.3278 | 3.4308 | 4.7586 | ↗ |
| *Prevotella dentalis* | S | 7 | 0.0306 | 2.0108 | 4.0662 | 2.0554 | ↗ |
| *Prevotella denticola* | S | 8 | 0.0306 | 1.4191 | 3.7995 | 2.3804 | ↗ |
| *Prevotella fusca* | S | 8 | 0.0306 | -0.7931 | 3.5596 | 4.3527 | ↗ |
| *Prevotella intermedia* | S | 10 | 0.0377 | 1.4585 | 3.8716 | 2.4131 | ↗ |
| *Prevotella jejuni* | S | 9 | 0.0360 | -1.298 | 3.2289 | 4.5269 | ↗ |
| *Prevotella melaninogenica* | S | 7 | 0.0306 | 2.0834 | 4.0574 | 1.974 | ↗ |
| *Prevotella sp. E13-17* | S | 6 | 0.0306 | -1.3307 | 3.4719 | 4.8026 | ↗ |
| *Prevotella sp. E13-3* | S | 7 | 0.0306 | -0.1956 | 4.0692 | 4.2648 | ↗ |
| *Prevotella sp. E9-3* | S | 12 | 0.0490 | 2.0063 | 3.8198 | 1.8135 | ↗ |
| *Prevotella sp. Rep29* | S | 12 | 0.0490 | 2.0851 | 3.9234 | 1.8383 | ↗ |
| *Priestia aryabhattai* | S | 105 | 0.0490 | 3.8677 | 0.7473 | -3.1204 | ↘ |
| *Priestia filamentosa* | S | 109 | 0.0306 | 2.3598 | -1.1211 | -3.4809 | ↘ |
| *Priestia flexa* | S | 108 | 0.0360 | 2.8454 | -0.4181 | -3.2635 | ↘ |
| *Proteiniphilum saccharofermentans* | S | 6 | 0.0306 | -0.857 | 3.3251 | 4.1821 | ↗ |
| *Pseudoprevotella muciniphila* | S | 6 | 0.0306 | -0.8453 | 3.5191 | 4.3644 | ↗ |
| *Radiobacillus kanasensis* | S | 105 | 0.0490 | 1.6657 | -1.4955 | -3.1612 | ↘ |
| *Ruminiclostridium herbifermentans* | S | 9 | 0.0360 | 1.7828 | 3.2005 | 1.4177 | ↗ |
| *Ruminococcus albus* | S | 7 | 0.0306 | 2.1515 | 4.0456 | 1.8941 | ↗ |
| *Ruminococcus bicirculans* | S | 0 | 0.0306 | 1.8491 | 4.2352 | 2.3861 | ↗ |
| *Ruminococcus bovis* | S | 0 | 0.0306 | -0.4106 | 4.3872 | 4.7978 | ↗ |
| *Sodaliphilus pleomorphus* | S | 6 | 0.0306 | 3.4652 | 6.1228 | 2.6576 | ↗ |
| *Solibacillus silvestris* | S | 109 | 0.0306 | 2.1935 | -1.1272 | -3.3207 | ↘ |
| *Staphylococcus aureus* | S | 105 | 0.0490 | 5.2442 | 3.7813 | -1.4629 | ↘ |
| *Streptococcus pasteurianus* | S | 5 | 0.0306 | -1.3966 | 2.7093 | 4.1059 | ↗ |
| *Tannerella serpentiformis* | S | 7 | 0.0306 | -0.3336 | 3.4142 | 3.7478 | ↗ |

¹P: Phylum; G: Genus; S: Species

²Statistic from Mann–Whitney U test

³P–value from Mann–Whitney U test after correction by Benjamini-Hochberg procedure

⁴↗: significantly increased abundance; ↘: significantly decreased abundance

Supplementary Table 3. Top 20 differentially abundant KOs from PPA and control samples

| KO¹ | statistic² | p_value³ | control mean relative abundance | ppa mean relative abundance | CLR mean difference | pathway_details⁴ | ko_name⁵ | ko_symbol⁶ |
| --- | --- | --- | --- | --- | --- | --- | --- | --- |
| ko:K16052 | 117 | 0.0365 | -1.1097 | 5.4692 | 6.5789 | NaN | MscS family membrane protein | ynaI |
| ko:K06228 | 106 | 0.0405 | -0.2867 | 5.5912 | 5.878 | Hedgehog signaling pathway - fly | fused | FU |
| ko:K01965 | 107 | 0.0405 | -0.2983 | 5.4543 | 5.7525 | Valine, Glyoxylate and dicarboxylate metabolism, Propanoate metabolism, Metabolic pathways, Biosynthesis of secondary metabolites, Microbial metabolism in diverse environments, Carbon metabolism | propionyl-CoA carboxylase alpha chain | PCCA |
| ko:K21279 | 103 | 0.0449 | -0.2924 | 5.3183 | 5.6107 | Amino sugar and nucleotide sugar metabolism, Metabolic pathways, Biosynthesis of nucleotide sugars | 3-deoxy-D-glycero-D-galacto-nononate 9-phosphate synthase | E2.5.1.132 |
| ko:K07783 | 104 | 0.043 | -0.3147 | 5.1548 | 5.4695 | Two-component system | MFS transporter | uhpC |
| ko:K07291 | 105 | 0.0405 | 0.2598 | 5.678 | 5.4182 | Inositol phosphate metabolism, Metabolic pathways | CDP-L-myo-inositol myo-inositolphosphotransferase | dipps |
| ko:K00091 | 103 | 0.0449 | 0.2141 | 5.1062 | 4.8921 | NaN | dihydroflavonol-4-reductase | E1.1.1.219 |
| ko:K00647 | 108 | 0.0405 | -0.4994 | 4.0118 | 4.5112 | Fatty acid biosynthesis, Biotin metabolism, Metabolic pathways, Fatty acid metabolism, Biosynthesis of cofactors | 3-oxoacyl- | fabB |
| ko:K03801 | 113 | 0.0405 | 2.156 | 6.3225 | 4.1665 | Lipoic acid metabolism, Metabolic pathways, Biosynthesis of cofactors | lipoyl(octanoyl) transferase | lipB |
| ko:K06143 | 105 | 0.0405 | -0.413 | 3.1955 | 3.6085 | NaN | inner membrane protein | creD |
| ko:K03390 | 117 | 0.0365 | -1.1097 | 2.4555 | 3.5651 | Methane metabolism, Metabolic pathways, Microbial metabolism in diverse environments, Carbon metabolism | heterodisulfide reductase subunit C2 | hdrC2 |
| ko:K03549 | 106 | 0.0405 | -0.4334 | 2.9939 | 3.4273 | NaN | KUP system potassium uptake protein | kup |
| ko:K15383 | 104 | 0.043 | -0.6913 | 2.696 | 3.3873 | NaN | MtN3 and saliva related transmembrane protein | K15383 |
| ko:K07492 | 109 | 0.0405 | 0.0708 | 3.417 | 3.3462 | NaN | putative transposase | K07492 |
| ko:K12343 | 108 | 0.0405 | -0.7049 | 2.6104 | 3.3153 | Steroid hormone biosynthesis, Metabolic pathways | 3-oxo-5-alpha-steroid 4-dehydrogenase 1 | SRD5A1 |
| ko:K01136 | 108 | 0.0405 | -0.7075 | 2.3779 | 3.0854 | Glycosaminoglycan degradation, Metabolic pathways, Lysosome | iduronate 2-sulfatase | IDS |
| ko:K06296 | 115 | 0.0365 | -0.5307 | 2.5309 | 3.0615 | NaN | spore germination protein KB | gerKB |
| ko:K06194 | 110 | 0.0405 | -0.7157 | 2.3113 | 3.027 | NaN | lipoprotein NlpD | nlpD |
| ko:K09516 | 106 | 0.0405 | -0.7318 | 2.1712 | 2.903 | Retinol metabolism | all-trans-retinol 13 | RETSAT |
| ko:K08364 | 105 | 0.0405 | -0.7037 | 2.1401 | 2.8438 | NaN | periplasmic mercuric ion binding protein | merP |
| ko:K21601 | 10 | 0.0405 | 4.1121 | -1.1848 | -5.2969 | NaN | DeoR family transcriptional regulator | ycnK |
| ko:K06342 | 10 | 0.0405 | 4.0042 | -1.1848 | -5.1889 | NaN | spore coat protein X | cotX |
| ko:K01569 | 10 | 0.0405 | 3.9423 | -1.1848 | -5.1271 | Glyoxylate and dicarboxylate metabolism, Metabolic pathways | oxalate decarboxylase | oxdD |
| ko:K07235 | 10 | 0.0405 | 3.9394 | -1.1848 | -5.1242 | Sulfur relay system | tRNA 2-thiouridine synthesizing protein D | tusD |
| ko:K18986 | 10 | 0.0405 | 3.9015 | -1.1848 | -5.0862 | Two-component system | two-component system | ihk |
| ko:K03297 | 10 | 0.0405 | 3.9 | -1.1848 | -5.0848 | NaN | small multidrug resistance pump | emrE |
| ko:K08713 | 10 | 0.0405 | 3.8946 | -1.1848 | -5.0794 | NaN | potassium channel LctB | lctB |
| ko:K00131 | 10 | 0.0405 | 3.8877 | -1.1848 | -5.0725 | Glycolysis / Gluconeogenesis, Pentose phosphate pathway, Metabolic pathways, Microbial metabolism in diverse environments, Carbon metabolism | glyceraldehyde-3-phosphate dehydrogenase (NADP+) | gapN |
| ko:K06418 | 10 | 0.0405 | 3.8801 | -1.1848 | -5.0648 | NaN | small acid-soluble spore protein A (major alpha-type SASP) | SASP-A |
| ko:K09118 | 10 | 0.0405 | 3.845 | -1.1848 | -5.0297 | NaN | uncharacterized protein | K09118 |
| ko:K22270 | 10 | 0.0405 | 3.8411 | -1.1848 | -5.0259 | Benzoate degradation, Microbial metabolism in diverse environments | 3-hydroxybenzoate 6-monooxygenase | nagX |
| ko:K18939 | 10 | 0.0405 | 3.8362 | -1.1848 | -5.0209 | NaN | TetR/AcrR family transcriptional regulator | lmrA |
| ko:K00368 | 10 | 0.0405 | 3.7708 | -1.1848 | -4.9555 | Nitrogen metabolism, Metabolic pathways, Microbial metabolism in diverse environments | nitrite reductase (NO-forming) | nirK |
| ko:K06417 | 10 | 0.0405 | 3.728 | -1.1848 | -4.9128 | NaN | stage VI sporulation protein D | spoVID |
| ko:K22074 | 9 | 0.0405 | 4.1193 | -0.2619 | -4.3812 | NaN | NFU1 iron-sulfur cluster scaffold homolog | NFU1 |
| ko:K06380 | 12 | 0.0405 | 3.1587 | -1.1848 | -4.3435 | NaN | stage II sporulation protein B | spoIIB |
| ko:K06436 | 12 | 0.0405 | 4.0866 | -0.208 | -4.2946 | NaN | spore coat assemly protein | yabG |
| ko:K06401 | 1 | 0.0365 | 3.4037 | -0.7495 | -4.1532 | NaN | stage IV sporulation protein FA | spoIVFA |
| ko:K07222 | 10 | 0.0405 | 2.5743 | -1.1848 | -3.759 | NaN | putative flavoprotein involved in K+ transport | K07222 |
| ko:K21832 | 13 | 0.043 | 3.4552 | -0.2544 | -3.7096 | Glycine, Metabolic pathways | glycine betaine monooxygenase B | gbcB |

¹KEGG Orthology ID

²Statistic from Mann–Whitney U test

³P–value from Mann–Whitney U test after correction by Benjamini-Hochberg procedure

⁴Pathways associated with the KO (NaN if none associated)

⁵Name(s) associated with KO (NaN if none associated)

⁶Symbol associated with KO (NaN if none associated)

Supplementary Table 4. Genera possessing predicted PPA metabolism and/or PPA production genes

| taxa | statistic¹ | p_value² | control mean relative abundance | PPA mean relative abundance | CLR mean difference | abundance in PPA³ | PPA metabolizing gene⁴ | PPA production gene⁵ |
| --- | --- | --- | --- | --- | --- | --- | --- | --- |
| *Acinetobacter* | 64 | 0.9090 | 4.259 | 4.1434 | -0.1156 | NS | ✔ |  |
| *Actinomadura* | 41 | 0.5939 | 0.6219 | 1.2138 | 0.5919 | NS | ✔ |  |
| *Acutalibacter* | 46 | 0.7320 | 6.3932 | 6.5991 | 0.2059 | NS | ✔ | ✔ |
| *Adlercreutzia* | 51 | 0.8931 | 3.4353 | 3.5474 | 0.1121 | NS | ✔ | ✔ |
| *Akkermansia* | 38 | 0.5097 | 7.2649 | 9.9488 | 2.6839 | NS | ✔ | ✔ |
| *Alkalihalobacillus* | 96 | 0.1570 | 3.2746 | 1.898 | -1.3766 | NS | ✔ |  |
| *Anaerobutyricum* | 36 | 0.4898 | 3.7313 | 4.0293 | 0.298 | NS | ✔ |  |
| *Anaerocolumna* | 44 | 0.6480 | 3.9063 | 4.136 | 0.2297 | NS | ✔ | ✔ |
| *Anaerostipes* | 51 | 0.8931 | 4.5707 | 4.6551 | 0.0844 | NS | ✔ |  |
| *Anaerotignum* | 46 | 0.7320 | 2.0337 | 2.7702 | 0.7365 | NS | ✔ |  |
| *Apibacter* | 50 | 0.8931 | -1.6533 | -1.1071 | 0.5462 | NS | ✔ |  |
| *Bacillus* | 95 | 0.1805 | 6.5113 | 5.5522 | -0.9591 | NS | ✔ | ✔ |
| *Bacteroides* | 11 | 0.0831 | 5.4028 | 6.7214 | 1.3186 | NS | ✔ | ✔ |
| *Bifidobacterium* | 37 | 0.5067 | 3.5332 | 3.7918 | 0.2586 | NS | ✔ |  |
| *Blautia* | 53 | 0.9090 | 6.9696 | 7.0539 | 0.0843 | NS | ✔ | ✔ |
| *Bradyrhizobium* | 38 | 0.5097 | 3.7471 | 4.0193 | 0.2722 | NS | ✔ | ✔ |
| *Brucella* | 34 | 0.4399 | 1.1116 | 2.1697 | 1.0581 | NS | ✔ |  |
| *Butyrivibrio* | 37 | 0.5067 | 4.576 | 4.79 | 0.214 | NS | ✔ |  |
| *Caldicellulosiruptor* | 30 | 0.3378 | 1.3212 | 2.1786 | 0.8574 | NS | ✔ |  |
| *Capnocytophaga* | 39 | 0.5339 | 2.2596 | 2.6022 | 0.3426 | NS | ✔ |  |
| *Caproicibacterium* | 6 | 0.0703 | 1.7155 | 3.5238 | 1.8083 | NS | ✔ | ✔ |
| *Caproiciproducens* | 29 | 0.3341 | 1.8273 | 2.5971 | 0.7698 | NS | ✔ |  |
| *Carnobacterium* | 65 | 0.9090 | 1.1789 | 0.7694 | -0.4095 | NS | ✔ | ✔ |
| *Christensenella* | 38 | 0.5097 | 3.0015 | 3.3735 | 0.372 | NS | ✔ |  |
| *Citrobacter* | 44 | 0.6480 | 3.1508 | 3.3363 | 0.1855 | NS | ✔ | ✔ |
| *Clavibacter* | 54 | 0.9090 | -2.7812 | -2.6469 | 0.1343 | NS |  | ✔ |
| *Clostridioides* | 56 | 0.9364 | 4.5039 | 4.5063 | 0.0024 | NS | ✔ |  |
| *Clostridium* | 58 | 1.0000 | 6.1663 | 6.2065 | 0.0402 | NS | ✔ | ✔ |
| *Coprococcus* | 48 | 0.8259 | 5.1602 | 5.3027 | 0.1425 | NS | ✔ | ✔ |
| *Corynebacterium* | 30 | 0.3378 | 4.1175 | 4.5251 | 0.4076 | NS | ✔ |  |
| *Crassaminicella* | 75 | 0.5939 | 1.5699 | 0.7348 | -0.8351 | NS | ✔ |  |
| *Cupriavidus* | 47 | 0.7752 | 3.7298 | 3.7931 | 0.0633 | NS | ✔ | ✔ |
| *Cytobacillus* | 90 | 0.2736 | 3.6504 | 2.7797 | -0.8707 | NS | ✔ |  |
| *Deinococcus* | 33 | 0.4062 | 2.472 | 2.8092 | 0.3372 | NS | ✔ |  |
| *Desulfobulbus* | 66 | 0.8931 | -2.2589 | -2.6049 | -0.346 | NS |  | ✔ |
| *Desulfuromonas* | 34 | 0.4399 | -1.1091 | -0.0665 | 1.0426 | NS | ✔ |  |
| *Devosia* | 55 | 0.9090 | 1.7408 | 2.0371 | 0.2963 | NS |  | ✔ |
| *Dolosigranulum* | 76 | 0.5939 | -1.308 | -3.0085 | -1.7005 | NS | ✔ |  |
| *Dorea* | 42 | 0.5939 | 4.2473 | 5.1082 | 0.8609 | NS | ✔ | ✔ |
| *Duncaniella* | 15 | 0.1196 | 5.2369 | 6.8608 | 1.6239 | NS | ✔ | ✔ |
| *Dysosmobacter* | 36 | 0.4898 | 5.541 | 6.0502 | 0.5092 | NS | ✔ | ✔ |
| *Echinicola* | 20 | 0.1406 | 1.3218 | 2.1032 | 0.7814 | NS | ✔ |  |
| *Eggerthella* | 37 | 0.5067 | 2.7708 | 3.1101 | 0.3393 | NS | ✔ |  |
| *Elizabethkingia* | 31 | 0.3597 | 1.9952 | 2.4186 | 0.4234 | NS | ✔ |  |
| *Enterobacter* | 37 | 0.5067 | 3.8575 | 4.1329 | 0.2754 | NS | ✔ | ✔ |
| *Enterocloster* | 55 | 0.9090 | 6.4065 | 6.5233 | 0.1168 | NS | ✔ | ✔ |
| *Enterococcus* | 71 | 0.7320 | 5.2696 | 5.0527 | -0.2169 | NS | ✔ | ✔ |
| *Erythrobacter* | 41 | 0.5939 | 0.0766 | 0.3917 | 0.3151 | NS | ✔ |  |
| *Escherichia* | 37 | 0.5067 | 5.22 | 5.6128 | 0.3928 | NS | ✔ |  |
| *Ethanoligenens* | 47 | 0.7752 | 1.996 | 2.6785 | 0.6825 | NS | ✔ |  |
| *Eubacterium* | 40 | 0.5670 | 4.69 | 4.9458 | 0.2558 | NS | ✔ | ✔ |
| *Evansella* | 100 | 0.1196 | 0.4279 | -3.0085 | -3.4364 | NS | ✔ |  |
| *Exiguobacterium* | 68 | 0.8496 | 1.9383 | 1.8991 | -0.0392 | NS | ✔ |  |
| *Faecalibacterium* | 42 | 0.5939 | 5.9106 | 6.1482 | 0.2376 | NS | ✔ | ✔ |
| *Faecalicatena* | 41 | 0.5939 | 3.1995 | 4.0121 | 0.8126 | NS | ✔ |  |
| *Flavonifractor* | 32 | 0.3794 | 5.8808 | 6.4169 | 0.5361 | NS | ✔ | ✔ |
| *Flintibacter* | 31 | 0.3597 | 4.963 | 5.5423 | 0.5793 | NS | ✔ | ✔ |
| *Gordonibacter* | 59 | 1.0000 | 1.2589 | 0.2695 | -0.9894 | NS | ✔ | ✔ |
| *Hungatella* | 68 | 0.8496 | 5.3546 | 5.3494 | -0.0052 | NS | ✔ |  |
| *Hydrogenophaga* | 24 | 0.2085 | 1.6868 | 2.6427 | 0.9559 | NS | ✔ | ✔ |
| *Intestinimonas* | 35 | 0.4627 | 4.7554 | 5.1919 | 0.4365 | NS | ✔ | ✔ |
| *Jeotgalibaca* | 63 | 0.9090 | 2.2546 | 1.912 | -0.3426 | NS | ✔ | ✔ |
| *Jeotgalicoccus* | 60 | 0.9663 | -1.2098 | -2.5972 | -1.3874 | NS | ✔ | ✔ |
| *Lachnoclostridium* | 55 | 0.9090 | 7.292 | 7.4125 | 0.1205 | NS | ✔ | ✔ |
| *Lachnospira* | 31 | 0.3597 | 2.03 | 2.8203 | 0.7903 | NS | ✔ | ✔ |
| *Lacrimispora* | 66 | 0.8931 | 4.9009 | 4.811 | -0.0899 | NS | ✔ | ✔ |
| *Lactiplantibacillus* | 46 | 0.7320 | -0.9557 | -0.4592 | 0.4965 | NS |  | ✔ |
| *Lederbergia* | 94 | 0.1942 | 1.0303 | -2.1772 | -3.2075 | NS | ✔ |  |
| *Leisingera* | 63 | 0.9090 | 1.5718 | 1.4252 | -0.1466 | NS | ✔ | ✔ |
| *Lentibacillus* | 107 | 0.0791 | 2.6002 | -1.4522 | -4.0524 | NS | ✔ |  |
| *Listeria* | 78 | 0.5339 | 3.3554 | 2.909 | -0.4464 | NS | ✔ |  |
| *Luteibacter* | 35 | 0.4627 | 0.6585 | 0.9767 | 0.3182 | NS | ✔ |  |
| *Lysinibacillus* | 82 | 0.4627 | 4.4183 | 3.4713 | -0.947 | NS | ✔ |  |
| *Mammaliicoccus* | 83 | 0.4399 | 6.07 | 4.3209 | -1.7491 | NS | ✔ | ✔ |
| *Marvinbryantia* | 45 | 0.6895 | 4.4391 | 5.3753 | 0.9362 | NS | ✔ | ✔ |
| *Massilistercora* | 52 | 0.9090 | 5.0897 | 5.1478 | 0.0581 | NS | ✔ | ✔ |
| *Methylomonas* | 49 | 0.8496 | 0.9446 | 1.4082 | 0.4636 | NS | ✔ |  |
| *Microbulbifer* | 14 | 0.1044 | 0.8073 | 2.2853 | 1.478 | NS | ✔ |  |
| *Microlunatus* | 76 | 0.5939 | -0.0634 | -1.1725 | -1.1091 | NS |  | ✔ |
| *Monoglobus* | 21 | 0.1570 | 0.7812 | 2.2147 | 1.4335 | NS | ✔ |  |
| *Mucilaginibacter* | 23 | 0.1942 | 2.6976 | 3.3012 | 0.6036 | NS | ✔ |  |
| *Muribaculum* | 17 | 0.1196 | 5.1853 | 6.7501 | 1.5648 | NS | ✔ | ✔ |
| *Neisseria* | 23 | 0.1942 | 2.3981 | 2.8542 | 0.4561 | NS | ✔ |  |
| *Nocardia* | 38 | 0.5097 | 2.7792 | 3.1276 | 0.3484 | NS | ✔ |  |
| *Novisyntrophococcus* | 32 | 0.3794 | 2.691 | 3.5675 | 0.8765 | NS |  | ✔ |
| *Novosphingobium* | 27 | 0.2736 | 1.629 | 2.5174 | 0.8884 | NS | ✔ |  |
| *Oceanidesulfovibrio* | 75 | 0.5939 | -2.1953 | -3.0085 | -0.8132 | NS | ✔ |  |
| *Oceanobacillus* | 99 | 0.1336 | 3.302 | 0.563 | -2.739 | NS | ✔ |  |
| *Oscillibacter* | 45 | 0.6895 | 4.2862 | 4.4972 | 0.211 | NS | ✔ | ✔ |
| *Oxalobacter* | 61 | 0.9364 | -1.6915 | -1.9171 | -0.2256 | NS | ✔ |  |
| *Paenibacillus* | 61 | 0.9364 | 6.7532 | 6.7447 | -0.0085 | NS | ✔ | ✔ |
| *Paeniclostridium* | 76 | 0.5939 | -1.6722 | -2.6469 | -0.9747 | NS | ✔ |  |
| *Pantoea* | 39 | 0.5339 | 2.8393 | 3.1056 | 0.2663 | NS | ✔ |  |
| *Paracoccus* | 30 | 0.3378 | 2.9859 | 3.3878 | 0.4019 | NS | ✔ |  |
| *Phocaeicola* | 19 | 0.1406 | 3.2899 | 4.4167 | 1.1268 | NS | ✔ |  |
| *Photobacterium* | 33 | 0.4062 | 1.5176 | 1.9574 | 0.4398 | NS | ✔ | ✔ |
| *Pluralibacter* | 40 | 0.5670 | -2.1819 | -1.1612 | 1.0207 | NS | ✔ |  |
| *Pontibacillus* | 97 | 0.1406 | 0.9345 | -2.6543 | -3.5888 | NS | ✔ |  |
| *Pontibacter* | 17 | 0.1196 | 0.8245 | 2.2439 | 1.4194 | NS | ✔ |  |
| *Priestia* | 93 | 0.2085 | 4.6286 | 3.4969 | -1.1317 | NS | ✔ |  |
| *Providencia* | 37 | 0.5067 | 1.9881 | 2.5375 | 0.5494 | NS | ✔ |  |
| *Pseudobutyrivibrio* | 30 | 0.3378 | 2.0025 | 2.9294 | 0.9269 | NS | ✔ |  |
| *Pseudochrobactrum* | 66 | 0.8931 | -2.2403 | -2.6543 | -0.414 | NS | ✔ |  |
| *Pseudomonas* | 38 | 0.5097 | 5.3737 | 5.645 | 0.2713 | NS | ✔ | ✔ |
| *Psychrobacter* | 43 | 0.6259 | -0.5131 | -0.0401 | 0.473 | NS | ✔ | ✔ |
| *Pusillibacter* | 35 | 0.4627 | 3.3086 | 4.3883 | 1.0797 | NS | ✔ | ✔ |
| *Qiania* | 46 | 0.7320 | 3.4265 | 4.213 | 0.7865 | NS | ✔ | ✔ |
| *Rhizobium* | 40 | 0.5670 | 3.7338 | 4.0601 | 0.3263 | NS | ✔ |  |
| *Roseburia* | 55 | 0.9090 | 6.3029 | 6.3605 | 0.0576 | NS | ✔ | ✔ |
| *Ruminococcus* | 13 | 0.0929 | 4.2849 | 5.0152 | 0.7303 | NS | ✔ | ✔ |
| *Ruthenibacterium* | 43 | 0.6259 | 3.7728 | 4.1047 | 0.3319 | NS | ✔ |  |
| *Salmonella* | 48 | 0.8259 | 3.8906 | 4.073 | 0.1824 | NS | ✔ |  |
| *Schaalia* | 44 | 0.6480 | 0.6337 | 0.8694 | 0.2357 | NS | ✔ |  |
| *Selenomonas* | 42 | 0.5939 | 2.3016 | 3.1084 | 0.8068 | NS | ✔ |  |
| *Sellimonas* | 38 | 0.5097 | 3.5122 | 4.4295 | 0.9173 | NS | ✔ | ✔ |
| *Serratia* | 37 | 0.5067 | 2.9817 | 3.2091 | 0.2274 | NS | ✔ |  |
| *Simiaoa* | 39 | 0.5339 | 5.0341 | 6.1217 | 1.0876 | NS | ✔ | ✔ |
| *Skermanella* | 68 | 0.8496 | 1.414 | 0.6275 | -0.7865 | NS | ✔ |  |
| *Slackia* | 79 | 0.5097 | -0.1082 | -1.5567 | -1.4485 | NS | ✔ |  |
| *Sodaliphilus* | 9 | 0.0791 | 2.166 | 4.6088 | 2.4428 | NS | ✔ |  |
| *Sphingomonas* | 38 | 0.5097 | 3.2875 | 3.6721 | 0.3846 | NS | ✔ |  |
| *Sporofaciens* | 57 | 0.9663 | 3.6597 | 4.4078 | 0.7481 | NS | ✔ | ✔ |
| *Sporosarcina* | 86 | 0.3597 | 5.0412 | 3.3314 | -1.7098 | NS | ✔ | ✔ |
| *Staphylococcus* | 91 | 0.2605 | 5.2262 | 4.2418 | -0.9844 | NS | ✔ |  |
| *Streptococcus* | 57 | 0.9663 | 4.9114 | 4.8163 | -0.0951 | NS | ✔ |  |
| *Streptomyces* | 41 | 0.5939 | 5.3457 | 5.7006 | 0.3549 | NS | ✔ | ✔ |
| *Subdoligranulum* | 27 | 0.2736 | 3.1639 | 4.234 | 1.0701 | NS | ✔ |  |
| *Thermomonas* | 32 | 0.3794 | -1.1719 | 0.0249 | 1.1968 | NS | ✔ |  |
| *Thomasclavelia* | 39 | 0.5339 | 4.6563 | 5.7403 | 1.084 | NS | ✔ | ✔ |
| *Vescimonas* | 33 | 0.4062 | 4.0768 | 5.1914 | 1.1146 | NS | ✔ | ✔ |
| *Vibrio* | 48 | 0.8259 | 4.1284 | 4.2836 | 0.1552 | NS | ✔ |  |
| *Virgibacillus* | 90 | 0.2736 | 4.0886 | 2.6611 | -1.4275 | NS | ✔ | ✔ |
| *Wansuia* | 44 | 0.6480 | 3.734 | 4.6164 | 0.8824 | NS | ✔ | ✔ |
| *Wujia* | 32 | 0.3794 | 2.578 | 3.4204 | 0.8424 | NS | ✔ |  |
| *Xanthomonas* | 38 | 0.5097 | 2.7684 | 3.0145 | 0.2461 | NS | ✔ |  |
| *Xiamenia* | 60 | 0.9663 | -2.7361 | -2.6402 | 0.0959 | NS | ✔ |  |

¹Statistic from Mann–Whitney U test

²P–value from Mann–Whitney U test after correction by Benjamini-Hochberg procedure

³NS: Non-significant difference in abundance

⁴✔: PPA metabolism gene present

⁵✔: PPA production gene present

Supplementary Table 5. Species possessing predicted PPA metabolism and/or PPA production genes

| taxa | statistic¹ | p_value² | control mean relative abundance | PPA mean relative abundance | CLR mean difference | abundance in PPA³ | PPA metabolizing gene⁴ | PPA production gene⁵ |
| --- | --- | --- | --- | --- | --- | --- | --- | --- |
| *Acutalibacter muris* | 43 | 0.5046 | 7.6844 | 8.1135 | 0.4291 | NS | ✔ | ✔ |
| *Adlercreutzia equolifaciens* | 47 | 0.5756 | 4.22 | 4.117 | -0.103 | NS | ✔ | ✔ |
| *Adlercreutzia hattorii* | 35 | 0.3608 | 3.8161 | 4.2221 | 0.406 | NS | ✔ | ✔ |
| *Akkermansia muciniphila* | 34 | 0.3305 | 8.5544 | 11.4631 | 2.9087 | NS | ✔ | ✔ |
| *Alkalihalobacillus clausii* | 76 | 0.5046 | 1.1166 | -0.3893 | -1.5059 | NS | ✔ |  |
| *Anaerobutyricum hallii* | 29 | 0.2037 | 5.0926 | 5.5432 | 0.4506 | NS | ✔ |  |
| *Anaerocolumna chitinilytica* | 37 | 0.4354 | 3.7125 | 4.664 | 0.9515 | NS |  | ✔ |
| *Anaerocolumna sedimenticola* | 41 | 0.5046 | 3.6325 | 4.5155 | 0.883 | NS | ✔ |  |
| *Anaerostipes caccae* | 56 | 0.9543 | 4.8942 | 5.1263 | 0.2321 | NS | ✔ |  |
| *Anaerostipes hadrus* | 39 | 0.4781 | 4.6019 | 4.9883 | 0.3864 | NS | ✔ |  |
| *Anaerostipes rhamnosivorans* | 46 | 0.5423 | 4.6944 | 5.0813 | 0.3869 | NS | ✔ |  |
| *Anaerotignum propionicum* | 37 | 0.4354 | 3.3057 | 4.285 | 0.9793 | NS | ✔ |  |
| *Bacillus aquiflavi* | 92 | 0.1682 | 1.7966 | -0.7368 | -2.5334 | NS | ✔ |  |
| *Bacillus cereus* | 98 | 0.0899 | 5.8987 | 3.9994 | -1.8993 | NS | ✔ | ✔ |
| *Bacillus cytotoxicus* | 85 | 0.2673 | 1.9522 | -0.2727 | -2.2249 | NS | ✔ | ✔ |
| *Bacillus mycoides* | 79 | 0.4680 | 3.4803 | 3.0125 | -0.4678 | NS | ✔ | ✔ |
| *Bacillus pseudomycoides* | 87 | 0.2168 | 2.0177 | -0.2875 | -2.3052 | NS | ✔ |  |
| *Bacillus shivajii* | 94 | 0.1453 | 2.1999 | -0.7538 | -2.9537 | NS | ✔ |  |
| *Bacillus subtilis* | 87 | 0.2168 | 5.1374 | 4.5815 | -0.5559 | NS | ✔ |  |
| *Bacillus thuringiensis* | 89 | 0.1881 | 3.8334 | 2.6351 | -1.1983 | NS | ✔ | ✔ |
| *Bacteroides caccae* | 36 | 0.3971 | 3.1937 | 3.8012 | 0.6075 | NS | ✔ |  |
| *Bacteroides cellulosilyticus* | 29 | 0.2037 | 4.1387 | 5.7778 | 1.6391 | NS | ✔ | ✔ |
| *Bacteroides nordii* | 4 | 0.0306 | 2.8439 | 6.0355 | 3.1916 | ↗ | ✔ | ✔ |
| *Bacteroides ovatus* | 5 | 0.0306 | 0.9192 | 4.6925 | 3.7733 | ↗ | ✔ |  |
| *Bacteroides zoogleoformans* | 13 | 0.0533 | 2.4991 | 4.7629 | 2.2638 | NS | ✔ | ✔ |
| *Bifidobacterium dentium* | 45 | 0.5046 | -1.4791 | -1.4955 | -0.0164 | NS | ✔ |  |
| *Blautia argi* | 41 | 0.5046 | 5.9726 | 6.3798 | 0.4072 | NS | ✔ | ✔ |
| *Blautia hansenii* | 38 | 0.4680 | 5.7845 | 6.2164 | 0.4319 | NS | ✔ | ✔ |
| *Blautia liquoris* | 65 | 0.7832 | 4.9858 | 5.1177 | 0.1319 | NS | ✔ |  |
| *Blautia obeum* | 53 | 0.8252 | 6.198 | 6.4774 | 0.2794 | NS | ✔ | ✔ |
| *Blautia producta* | 53 | 0.8252 | 6.3198 | 6.5886 | 0.2688 | NS | ✔ | ✔ |
| *Blautia pseudococcoides* | 57 | 0.9874 | 6.1516 | 6.3998 | 0.2482 | NS | ✔ | ✔ |
| *Blautia wexlerae* | 51 | 0.7460 | 6.2298 | 6.5214 | 0.2916 | NS | ✔ | ✔ |
| *Butyrivibrio fibrisolvens* | 32 | 0.2673 | 4.6133 | 5.0445 | 0.4312 | NS | ✔ |  |
| *Butyrivibrio hungatei* | 44 | 0.5046 | 4.228 | 4.6367 | 0.4087 | NS | ✔ |  |
| *Butyrivibrio proteoclasticus* | 51 | 0.7460 | 4.4602 | 4.6933 | 0.2331 | NS | ✔ |  |
| *Capnocytophaga canimorsus* | 45 | 0.5046 | -1.4685 | -1.4955 | -0.027 | NS | ✔ |  |
| *Clostridium baratii* | 55 | 0.9068 | 0.2548 | -0.0473 | -0.3021 | NS | ✔ |  |
| *Clostridium botulinum* | 40 | 0.5046 | 4.5707 | 4.8253 | 0.2546 | NS | ✔ | ✔ |
| *Clostridium estertheticum* | 72 | 0.5046 | 2.969 | 2.7574 | -0.2116 | NS | ✔ | ✔ |
| *Clostridium hylemonae* | 59 | 1.0000 | 5.8859 | 6.0614 | 0.1755 | NS | ✔ | ✔ |
| *Clostridium innocuum* | 50 | 0.7110 | 6.4962 | 6.7683 | 0.2721 | NS | ✔ | ✔ |
| *Clostridium intestinale* | 92 | 0.1682 | 0.7058 | -1.4955 | -2.2013 | NS | ✔ |  |
| *Clostridium novyi* | 52 | 0.7832 | -0.9674 | -1.1339 | -0.1665 | NS | ✔ |  |
| *Clostridium scindens* | 51 | 0.7460 | 7.3737 | 7.6789 | 0.3052 | NS | ✔ | ✔ |
| *Coprococcus catus* | 36 | 0.3971 | 5.0202 | 5.4236 | 0.4034 | NS | ✔ | ✔ |
| *Coprococcus comes* | 41 | 0.5046 | 5.4585 | 5.8514 | 0.3929 | NS | ✔ | ✔ |
| *Cupriavidus taiwanensis* | 32 | 0.2673 | 2.0926 | 2.5293 | 0.4367 | NS | ✔ | ✔ |
| *Cytobacillus oceanisediminis* | 102 | 0.0647 | 3.5009 | 0.5015 | -2.9994 | NS | ✔ |  |
| *Dolosigranulum pigrum* | 70 | 0.5756 | 0.0041 | -1.4955 | -1.4996 | NS | ✔ |  |
| *Dorea formicigenerans* | 53 | 0.8252 | 5.2686 | 5.4334 | 0.1648 | NS | ✔ |  |
| *Dorea longicatena* | 52 | 0.7832 | 6.0076 | 6.2564 | 0.2488 | NS | ✔ | ✔ |
| *Duncaniella dubosii* | 7 | 0.0306 | 5.8039 | 7.5896 | 1.7857 | ↗ | ✔ | ✔ |
| *Dysosmobacter welbionis* | 34 | 0.3305 | 6.2447 | 6.8437 | 0.599 | NS | ✔ | ✔ |
| *Eggerthella lenta* | 47 | 0.5756 | 3.7028 | 4.0705 | 0.3677 | NS | ✔ |  |
| *Enterobacter cloacae* | 48 | 0.6194 | 2.9782 | 3.4844 | 0.5062 | NS | ✔ |  |
| *Enterocloster bolteae* | 49 | 0.6664 | 7.1473 | 7.463 | 0.3157 | NS | ✔ | ✔ |
| *Enterocloster clostridioformis* | 44 | 0.5046 | 5.8906 | 6.2742 | 0.3836 | NS | ✔ | ✔ |
| *Enterococcus cecorum* | 88 | 0.2037 | 2.9599 | 1.5615 | -1.3984 | NS | ✔ |  |
| *Enterococcus faecalis* | 41 | 0.5046 | 5.7801 | 5.9735 | 0.1934 | NS | ✔ |  |
| *Enterococcus faecium* | 51 | 0.7460 | 5.3738 | 5.2496 | -0.1242 | NS | ✔ | ✔ |
| *Ethanoligenens harbinense* | 30 | 0.2168 | 3.2849 | 4.1941 | 0.9092 | NS | ✔ |  |
| *Eubacterium callanderi* | 42 | 0.5046 | 2.4184 | 3.0922 | 0.6738 | NS | ✔ |  |
| *Eubacterium hominis* | 14 | 0.0618 | 3.9786 | 4.6083 | 0.6297 | NS | ✔ |  |
| *Eubacterium ventriosum* | 58 | 1.0000 | 3.8604 | 3.6924 | -0.168 | NS | ✔ |  |
| *Evansella cellulosilytica* | 92 | 0.1682 | 0.9291 | -1.4955 | -2.4246 | NS | ✔ |  |
| *Faecalibacterium duncaniae* | 25 | 0.1682 | 5.657 | 6.2426 | 0.5856 | NS | ✔ |  |
| *Faecalibacterium prausnitzii* | 42 | 0.5046 | 6.4697 | 6.8941 | 0.4244 | NS | ✔ | ✔ |
| *Flavonifractor plautii* | 23 | 0.1453 | 7.188 | 7.9313 | 0.7433 | NS | ✔ | ✔ |
| *Gordonibacter pamelaeae* | 39 | 0.4781 | -0.8453 | 0.3639 | 1.2092 | NS | ✔ | ✔ |
| *Hungatella hathewayi* | 67 | 0.7110 | 6.7245 | 6.864 | 0.1395 | NS | ✔ |  |
| *Intestinimonas butyriciproducens* | 23 | 0.1453 | 6.0378 | 6.707 | 0.6692 | NS | ✔ | ✔ |
| *Jeotgalibaca arthritidis* | 71 | 0.5423 | -0.0702 | -1.4955 | -1.4253 | NS | ✔ |  |
| *Jeotgalibaca dankookensis* | 65 | 0.7832 | -0.0578 | -1.1211 | -1.0633 | NS | ✔ |  |
| *Jeotgalibaca porci* | 45 | 0.5046 | -1.1891 | -1.4955 | -0.3064 | NS | ✔ | ✔ |
| *Jeotgalicoccus saudimassiliensis* | 58 | 1.0000 | -0.1708 | -1.4955 | -1.3247 | NS | ✔ | ✔ |
| *Lachnoclostridium phocaeense* | 55 | 0.9068 | 6.8112 | 7.0719 | 0.2607 | NS | ✔ | ✔ |
| *Lachnospira eligens* | 27 | 0.1817 | 3.2735 | 4.3333 | 1.0598 | NS | ✔ | ✔ |
| *Lacrimispora saccharolytica* | 64 | 0.8252 | 4.9612 | 5.2488 | 0.2876 | NS | ✔ |  |
| *Lacrimispora xylanolytica* | 62 | 0.9068 | 4.9041 | 5.086 | 0.1819 | NS | ✔ | ✔ |
| *Lactiplantibacillus plantarum* | 45 | 0.5046 | -1.317 | -1.4955 | -0.1785 | NS |  | ✔ |
| *Lederbergia lenta* | 94 | 0.1453 | 2.3343 | -0.6641 | -2.9984 | NS | ✔ |  |
| *Listeria ivanovii* | 87 | 0.2168 | 1.1931 | -1.4955 | -2.6886 | NS | ✔ |  |
| *Luteibacter rhizovicinus* | 45 | 0.5046 | -1.4685 | -1.4955 | -0.027 | NS | ✔ |  |
| *Mammaliicoccus lentus* | 86 | 0.2391 | 7.2559 | 3.8536 | -3.4023 | NS | ✔ | ✔ |
| *Mammaliicoccus sciuri* | 86 | 0.2391 | 3.8063 | 1.597 | -2.2093 | NS | ✔ |  |
| *Marvinbryantia formatexigens* | 57 | 0.9874 | 6.6388 | 6.8901 | 0.2513 | NS | ✔ | ✔ |
| *Massilistercora timonensis* | 55 | 0.9068 | 6.3859 | 6.6621 | 0.2762 | NS | ✔ | ✔ |
| *Monoglobus pectinilyticus* | 11 | 0.0456 | 2.0833 | 3.7277 | 1.6444 | ↗ | ✔ |  |
| *Muribaculum gordoncarteri* | 13 | 0.0533 | 5.9127 | 7.5464 | 1.6337 | NS | ✔ | ✔ |
| *Muribaculum intestinale* | 7 | 0.0306 | 5.6228 | 7.5802 | 1.9574 | ↗ | ✔ | ✔ |
| *Novisyntrophococcus fermenticellae* | 39 | 0.4781 | 4.6754 | 5.0831 | 0.4077 | NS |  | ✔ |
| *Oceanidesulfovibrio marinus* | 58 | 1.0000 | -0.8932 | -1.4955 | -0.6023 | NS | ✔ |  |
| *Oceanobacillus jeddahense* | 102 | 0.0647 | 2.7483 | -1.1272 | -3.8755 | NS | ✔ |  |
| *Oceanobacillus oncorhynchi* | 100 | 0.0706 | 1.9318 | -1.4955 | -3.4273 | NS | ✔ |  |
| *Oceanobacillus zhaokaii* | 100 | 0.0706 | 2.967 | -0.7471 | -3.7141 | NS | ✔ |  |
| *Oscillibacter hominis* | 34 | 0.3305 | 5.4411 | 6.0122 | 0.5711 | NS | ✔ | ✔ |
| *Oxalobacter vibrioformis* | 45 | 0.5046 | -1.4791 | -1.4955 | -0.0164 | NS | ✔ |  |
| *Paenibacillus albus* | 47 | 0.5756 | -0.9194 | -0.7558 | 0.1636 | NS | ✔ |  |
| *Paenibacillus donghaensis* | 45 | 0.5046 | 3.5927 | 3.9173 | 0.3246 | NS | ✔ |  |
| *Paeniclostridium sordellii* | 62 | 0.9068 | -0.3701 | -1.1339 | -0.7638 | NS | ✔ |  |
| *Paracoccus denitrificans* | 45 | 0.5046 | -1.4588 | -1.4955 | -0.0367 | NS | ✔ |  |
| *Phocaeicola salanitronis* | 26 | 0.1817 | 2.3705 | 3.9869 | 1.6164 | NS | ✔ |  |
| *Pluralibacter gergoviae* | 34 | 0.3305 | -1.434 | -0.3912 | 1.0428 | NS | ✔ |  |
| *Priestia megaterium* | 83 | 0.3305 | 5.4054 | 4.5774 | -0.828 | NS | ✔ |  |
| *Pseudobutyrivibrio xylanivorans* | 18 | 0.0794 | 3.3194 | 4.4424 | 1.123 | NS | ✔ |  |
| *Pseudochrobactrum algeriensis* | 58 | 1.0000 | -0.9568 | -1.4955 | -0.5387 | NS | ✔ |  |
| *Pseudomonas sp.* | 45 | 0.5046 | -1.4685 | -1.4955 | -0.027 | NS | ✔ | ✔ |
| *Pusillibacter faecalis* | 35 | 0.3608 | 5.3582 | 5.9021 | 0.5439 | NS | ✔ | ✔ |
| *Qiania dongpingensis* | 60 | 0.9874 | 5.4892 | 5.7272 | 0.238 | NS | ✔ | ✔ |
| *Roseburia hominis* | 57 | 0.9874 | 7.0828 | 7.3586 | 0.2758 | NS | ✔ | ✔ |
| *Roseburia intestinalis* | 58 | 1.0000 | 6.3387 | 6.6177 | 0.279 | NS | ✔ | ✔ |
| *Ruminococcus bicirculans* | 0 | 0.0306 | 1.8491 | 4.2352 | 2.3861 | ↗ | ✔ |  |
| *Ruminococcus bovis* | 0 | 0.0306 | -0.4106 | 4.3872 | 4.7978 | ↗ | ✔ | ✔ |
| *Ruminococcus gauvreauii* | 40 | 0.5046 | 5.1705 | 5.5752 | 0.4047 | NS | ✔ |  |
| *Ruminococcus gnavus* | 65 | 0.7832 | 6.8723 | 7.0238 | 0.1515 | NS | ✔ | ✔ |
| *Ruminococcus lactaris* | 46 | 0.5423 | 5.1201 | 5.4624 | 0.3423 | NS | ✔ |  |
| *Ruthenibacterium lactatiformans* | 29 | 0.2037 | 5.0685 | 5.6205 | 0.552 | NS | ✔ |  |
| *Salmonella enterica* | 40 | 0.5046 | 5.1063 | 5.4987 | 0.3924 | NS | ✔ |  |
| *Selenomonas sputigena* | 45 | 0.5046 | 2.0849 | 2.4687 | 0.3838 | NS | ✔ |  |
| *Sellimonas intestinalis* | 45 | 0.5046 | 5.596 | 5.9457 | 0.3497 | NS | ✔ | ✔ |
| *Serratia liquefaciens* | 39 | 0.4781 | -1.4685 | -1.1339 | 0.3346 | NS | ✔ |  |
| *Simiaoa sunii* | 49 | 0.6664 | 7.3213 | 7.6362 | 0.3149 | NS | ✔ | ✔ |
| *Slackia heliotrinireducens* | 73 | 0.5046 | 1.2015 | -0.0437 | -1.2452 | NS | ✔ |  |
| *Sodaliphilus pleomorphus* | 6 | 0.0306 | 3.4652 | 6.1228 | 2.6576 | ↗ | ✔ |  |
| *Sporofaciens musculi* | 71 | 0.5423 | 5.7701 | 5.922 | 0.1519 | NS | ✔ | ✔ |
| *Sporosarcina pasteurii* | 92 | 0.1682 | 4.584 | 0.8116 | -3.7724 | NS | ✔ | ✔ |
| *Sporosarcina ureae* | 86 | 0.2391 | 3.5252 | 0.5082 | -3.017 | NS | ✔ |  |
| *Sporosarcina ureilytica* | 82 | 0.3608 | 4.1934 | 0.8453 | -3.3481 | NS | ✔ | ✔ |
| *Staphylococcus nepalensis* | 58 | 1.0000 | -0.7721 | -1.4955 | -0.7234 | NS | ✔ |  |
| *Staphylococcus simulans* | 100 | 0.0706 | 1.6711 | -1.4955 | -3.1666 | NS | ✔ |  |
| *Streptococcus acidominimus* | 83 | 0.3305 | 0.1188 | -1.4955 | -1.6143 | NS | ✔ |  |
| *Streptococcus anginosus* | 54 | 0.8618 | -0.8298 | -1.4955 | -0.6657 | NS | ✔ |  |
| *Streptococcus dysgalactiae* | 78 | 0.4781 | 2.6578 | 1.1357 | -1.5221 | NS | ✔ |  |
| *Streptococcus pluranimalium* | 45 | 0.5046 | -1.4077 | -1.4955 | -0.0878 | NS | ✔ |  |
| *Streptococcus urinalis* | 45 | 0.5046 | -1.3647 | -1.4955 | -0.1308 | NS | ✔ |  |
| *Subdoligranulum variabile* | 31 | 0.2391 | 5.2067 | 5.7479 | 0.5412 | NS | ✔ |  |
| *Thomasclavelia ramosa* | 40 | 0.5046 | 4.1177 | 5.3487 | 1.231 | NS | ✔ | ✔ |
| *Thomasclavelia spiroformis* | 39 | 0.4781 | 3.675 | 4.8699 | 1.1949 | NS | ✔ | ✔ |
| *Vescimonas coprocola* | 34 | 0.3305 | 5.5908 | 6.1186 | 0.5278 | NS | ✔ | ✔ |
| *Vescimonas fastidiosa* | 37 | 0.4354 | 5.4404 | 5.8907 | 0.4503 | NS | ✔ |  |
| *Virgibacillus necropolis* | 104 | 0.0533 | 3.0806 | -1.4955 | -4.5761 | NS | ✔ | ✔ |
| *Virgibacillus pantothenticus* | 97 | 0.1002 | 3.3321 | -0.227 | -3.5591 | NS | ✔ | ✔ |
| *Virgibacillus phasianinus* | 97 | 0.1002 | 2.2912 | -1.1272 | -3.4184 | NS | ✔ | ✔ |
| *Wansuia hejianensis* | 56 | 0.9543 | 5.8377 | 6.1309 | 0.2932 | NS | ✔ | ✔ |
| *Wujia chipingensis* | 36 | 0.3971 | 4.5405 | 4.9346 | 0.3941 | NS | ✔ |  |
| *Xiamenia xianingshaonis* | 43 | 0.5046 | -1.434 | -1.1272 | 0.3068 | NS | ✔ |  |

¹Statistic from Mann–Whitney U test

²P–value from Mann–Whitney U test after correction by Benjamini-Hochberg procedure

³↗: Non-significant difference in abundance; significantly increased abundance; ↘: significantly decreased abundance in PPA samples

⁴✔: PPA metabolism gene present

⁵✔: PPA production gene present

Supplementary Table 6. Predicted PPA metabolizing/producing species with significantly shifted abundances

| taxa | statistic¹ | p_value² | control mean relative abundance | PPA mean relative abundance | CLR mean difference | abundance in PPA³ | ppa metabolizing gene⁴ | PPA production gene⁵ |
| --- | --- | --- | --- | --- | --- | --- | --- | --- |
| *Bacteroides nordii* | 4 | 0.0306 | 2.8439 | 6.0355 | 3.1916 | ↗ | ✔ | ✔ |
| *Bacteroides ovatus* | 5 | 0.0306 | 0.9192 | 4.6925 | 3.7733 | ↗ | ✔ |  |
| *Duncaniella dubosii* | 7 | 0.0306 | 5.8039 | 7.5896 | 1.7857 | ↗ | ✔ | ✔ |
| *Monoglobus pectinilyticus* | 11 | 0.0456 | 2.0833 | 3.7277 | 1.6444 | ↗ | ✔ |  |
| *Muribaculum intestinale* | 7 | 0.0306 | 5.6228 | 7.5802 | 1.9574 | ↗ | ✔ | ✔ |
| *Ruminococcus bicirculans* | 0 | 0.0306 | 1.8491 | 4.2352 | 2.3861 | ↗ | ✔ |  |
| *Ruminococcus bovis* | 0 | 0.0306 | -0.4106 | 4.3872 | 4.7978 | ↗ | ✔ | ✔ |
| *Sodaliphilus pleomorphus* | 6 | 0.0306 | 3.4652 | 6.1228 | 2.6576 | ↗ | ✔ |  |

¹Statistic from Mann–Whitney U test

²P–value from Mann–Whitney U test after correction by Benjamini-Hochberg procedure

³↗: significantly increased abundance; ↘: significantly decreased abundance in PPA samples

⁴✔: PPA metabolism gene present

⁵✔: PPA production gene present
